# Microbial Diversity and Function in Shallow Subsurface Sediment and Oceanic Lithosphere of the Atlantis Massif

**DOI:** 10.1128/mBio.00490-21

**Published:** 2021-08-03

**Authors:** J. Goordial, T. D’Angelo, J. M. Labonté, N. J. Poulton, J. M. Brown, R. Stepanauskas, G. L. Früh-Green, B. N. Orcutt

**Affiliations:** a Bigelow Laboratory for Ocean Sciences, East Boothbay, Maine, USA; b University of Guelph, Guelph, Ontario, Canada; c Texas A&M University at Galveston, Galveston, Texas, USA; d ETH Zürich, Zürich, Switzerland; e Hanse Institute for Advanced Study, Delmenhorst, Germany; Max Planck Institute for Marine Microbiology

**Keywords:** Atlantis Massif, deep biosphere, oceanic crust, IODP, single-cell genomics, fluorescence-activated cell sorting, lithosphere

## Abstract

The marine lithospheric subsurface is one of the largest biospheres on Earth; however, little is known about the identity and ecological function of microorganisms found in low abundance in this habitat, though these organisms impact global-scale biogeochemical cycling. Here, we describe the diversity and metabolic potential of sediment and endolithic (within rock) microbial communities found in ultrasmall amounts (10^1^ to 10^4^ cells cm^−3^) in the subsurface of the Atlantis Massif, an oceanic core complex on the Mid-Atlantic Ridge that was sampled on International Ocean Discovery Program (IODP) Expedition 357. This study used fluorescence-activated cell sorting (FACS) to enable the first amplicon, metagenomic, and single-cell genomic study of the shallow (<20 m below seafloor) subsurface of an actively serpentinizing marine system. The shallow subsurface biosphere of the Atlantis Massif was found to be distinct from communities observed in the nearby Lost City alkaline hydrothermal fluids and chimneys, yet similar to other low-temperature, aerobic subsurface settings. Genes associated with autotrophy were rare, although heterotrophy and aerobic carbon monoxide and formate cycling metabolisms were identified. Overall, this study reveals that the shallow subsurface of an oceanic core complex hosts a biosphere that is not fueled by active serpentinization reactions and by-products.

## INTRODUCTION

Much of the recent investigations into the marine deep biosphere have focused on microbial life in deep sediments, which has revealed life to be extensive, diverse, and capable of maintaining slow metabolic activity on long timescales (1–3). Less is known about the microbial life hosted within the oceanic lithosphere. In spite of generally low biomass in the oceanic lithosphere, given the sheer volume of this habitat, crustal microbiota can impact global biogeochemical cycling, and the marine rock subsurface is potentially one of the largest biospheres on Earth (4, 5), and crustal microbiota can impact global biogeochemical cycling. For example, crustal microorganisms may be responsible for removing at least 5% of global ocean dissolved organic carbon (6). The diversity, metabolism, and potential rate of activity of microorganisms hosted within oceanic crust remains poorly constrained. The potential for both autotrophy and heterotrophy has been detected in crustal environments in activity assays in bulk samples or by accessing the fluids moving through the crust via subsurface observatories (7, 8), but these processes are poorly resolved. Currently, our understanding of the crustal deep biosphere is informed from studies of rocks at only a few sites on Earth, which revealed diverse microbial community structures, as reviewed elsewhere (9). Linking biogeochemical cycling with phylogeny and genetics of endolithic microorganisms through molecular analyses remains challenging, due to the low biomass in the crustal subsurface and difficulties in extracting DNA from these environmental samples (10–13).

The Atlantis Massif is an underwater mountain on the flank of the Mid-Atlantic Ridge at 30°N, where ultramafic and mafic rock sequences have been uplifted to the seafloor along a major detachment fault zone in an oceanic core complex. Host to the Lost City Hydrothermal Field (LCHF) along its southern wall, the Atlantis Massif is a site of active serpentinization, where oxidized seawater interacts with olivine-rich rock to produce serpentinite in the subsurface (14). Serpentinization reactions create alkaline conditions (pH 9 to 12) and result in the abiotic production of H_2_, CH_4_, formate, and other short-chain alkanes (15–18). These by-products can be used by microorganisms for carbon and energy in the subsurface. For example, LCHF vent fluids have large amounts of methane, supporting low diversity, methane-cycling *Archaea*, sulfur-oxidizing *Bacteria*, and formate utilization (16, 19–23). While the LCHF represents a unique, high-pH microbial community, the nature of the biosphere within the crustal core of the Atlantis Massif is unknown. The central dome of the Atlantis Massif was previously drilled during Integrated Ocean Drilling Program Expeditions 304/305, but this gabbroic-rich section was likely not representative of the crustal conditions underlying Lost City (14).

**FIG 1:**
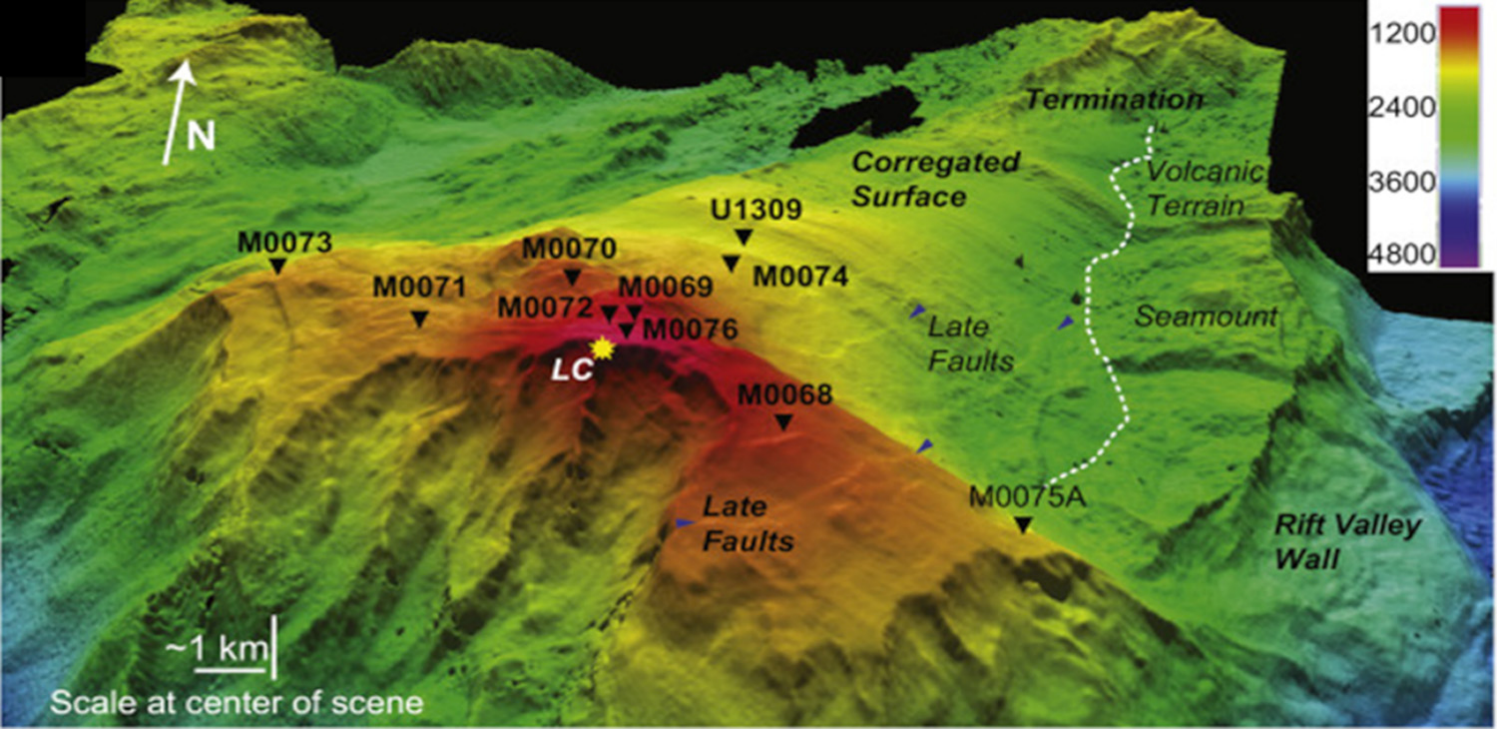
Bathymetric map showing IODP Expedition 357 sites where samples were collected. The color legend represents water depth in meters. The figure was reproduced from Früh-Green et al. (14) with permission.

To investigate the nature of the deep biosphere in the serpentinizing oceanic core complex of the Atlantis Massif, International Ocean Discovery Program (IODP) Expedition 357 drilled 17 shallow holes at nine sites across the southern wall of the massif (14) (Fig. 1). The 57 m of recovered cores were highly heterogeneous, including pervasively serpentinized ultramafic sequences as well as mafic units of gabbros, dolerites, and basalts that record multiple phases of magmatism, fluid-rock interaction, and mass transfer (14, 24). Approximately 8 m was subsampled for microbiological investigation (25, 26). Microbial cell densities in the core samples were very low, ranging from 10^1^ to 10^4^ cells cm^−3^ (14).

**FIG 2:**
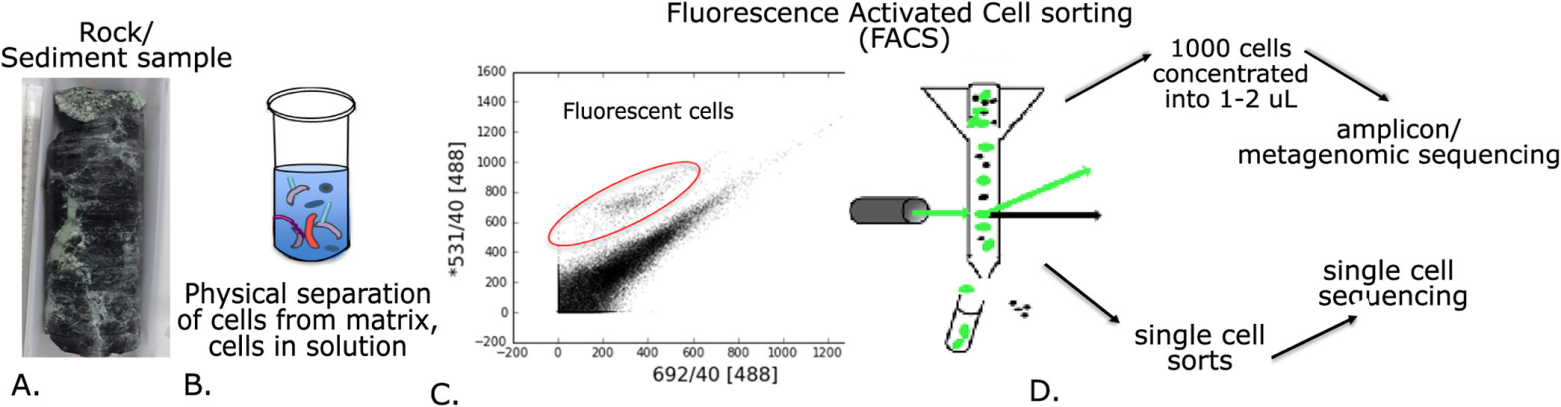
Overview of fluorescence-activated cell sorting (FACS) method via flow cytometry for cell concentration of low biomass samples. (A) Low biomass environmental samples of rock and sediment from IODP Expedition 357. (B) Samples were gently sonicated and vortexed in a salt buffer to physically separate cells from the substrate and suspend in liquid solution. (C) Cells in the supernatant were stained with a fluorescent general DNA stain (SYTO green) and subjected to FACS, with cells identified via fluorescent particle properties and size. (D) Cell-like particles were either separated individually or pooled as 1,000 cells into 1 to 2 μl of a salt buffer landing pad. Single-cell sorts and 1,000-cell sorts were subjected to downstream genomic analyses after whole-genome amplification via multiple displacement amplification.

To overcome the limitations of bulk DNA extraction from low biomass crustal samples, which is detailed extensively for this site (27), we used fluorescence-activated cell sorting (FACS) as a means to concentrate cells from the Atlantis Massif samples to enable various downstream DNA sequencing (27, 28) (Fig. 2). From these cell sorts, we queried microbial community diversity and function to examine how this actively serpentinizing, shallow, ultramafic ecosystem compares to the LCHF and other deep crustal environments. This cell sorting-based approach enabled more genomic information to be recovered from this site than has been possible with prior bulk nucleic acid extraction approaches, indicating the promise of this approach for other low biomass investigations.

## RESULTS AND DISCUSSION

### Heterogeneous microbial community composition in the shallow subsurface of the Atlantis Massif.

**FIG 3:**
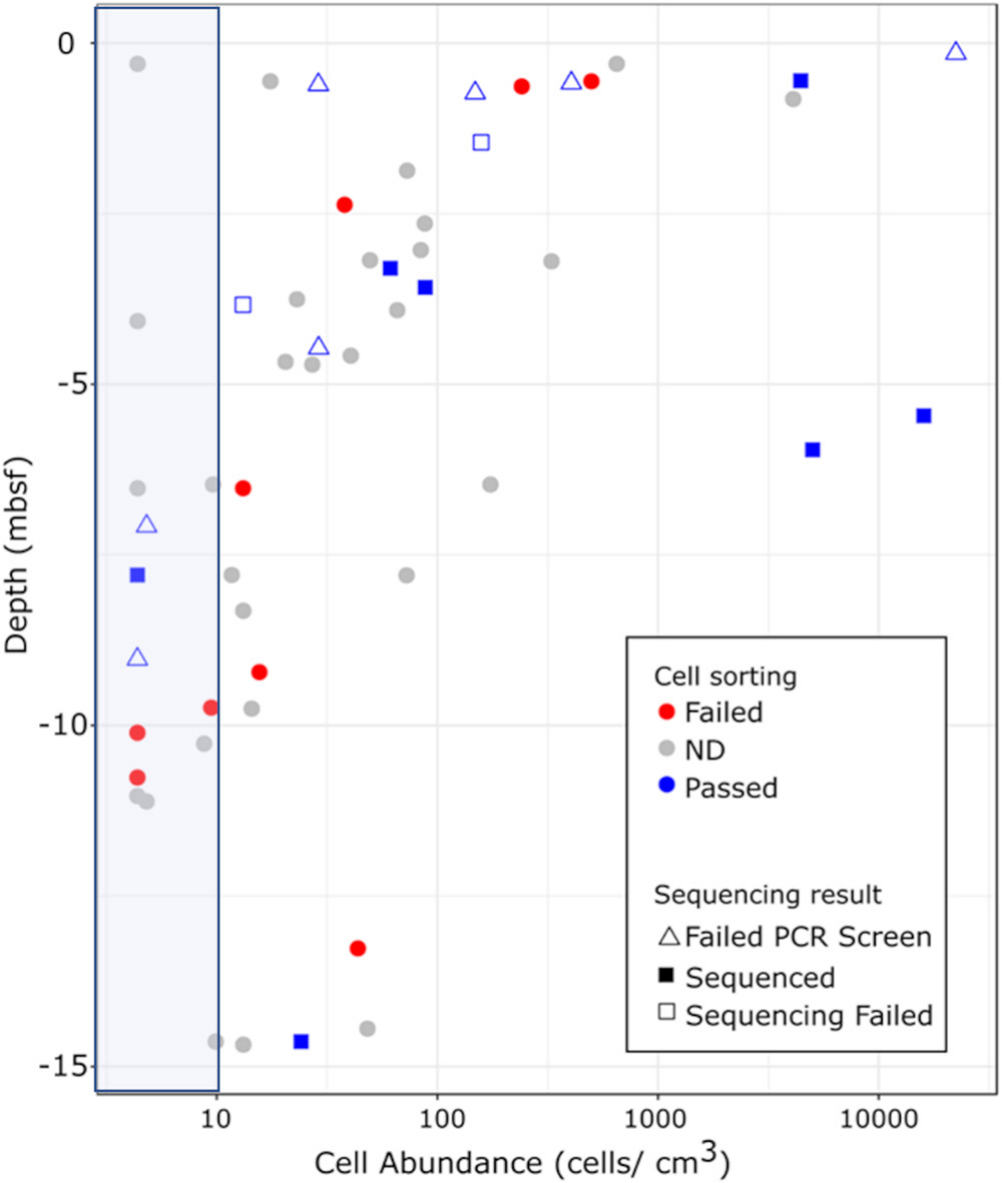
Success of cell sorting and DNA amplification/sequencing in Atlantis Massif shallow subsurface samples as a function of sample cell concentration and depth in meters below seafloor (mbsf). Cell abundance data are from Früh-Green et al. (14) on parallel subsamples, and the shaded band represents the limits of cell quantification (<10 cells per cubic cm) in that study. Red filled circles labeled “Failed” in the symbol legend show that no cells were detected on the flow cytometer. Gray filled circles labeled “ND” show parallel samples for which cell density data exist in Früh-Green et al. (14) but that were not analyzed in this study. Open blue triangles labeled “Failed PCR screen” represent samples that had detectable cells in the flow cytometer but amplified DNA extracts from sorted cells did not have an observable PCR product when run on an agarose gel. Open blue squares labeled “Sequencing Failed” represent samples that had detectable cells in the flow cytometer and amplified DNA extracts from sorted cells had an observable PCR product, but amplicon sequencing failed. Filled blue squares labeled “Sequenced” represent samples that had detectable cells on the flow cytometer, and 16S rRNA genes were successfully sequenced from amplified DNA extracts from pooled sorted cells. The observation that both successful cell sorting (all blue symbols) and sequencing success (blue filled squares) did not correlate with cell density indicates that the gentle cell extraction approach used in the study was not biased by cell density.

A total of 29 sediment and crustal samples were screened with the bulk cell sorting approach (Table 1), with 18 yielding cell-like particles (Fig. 3 and Table 1). Even with the steps taken to increase cell concentration (i.e., extracting cells from several grams of rock and concentrating to a few milliliters of fluid), samples took between 30 to 60 min of sorting to collect a sufficient number of cells (when no cells were detected, the flow cytometer was run until samples were exhausted). Sufficient DNA for amplification and amplicon sequencing of the 16S rRNA gene was successfully obtained from 8 out of the 18 samples. Three of these eight were from sediment samples, and the other five were from crust samples. Comparison of the cell detection and sequencing success of this study with the cell densities determined in parallel samples (14) indicates that the limit of this methodology does not correspond with cell density. Based on this sample set, the approach of extracting and concentrating cells via FACS from <10 g of low biomass sediment and crust samples to yield sufficient DNA for 16S rRNA gene amplicon sequencing had an overall efficiency of 8 out of 29 samples (28%). While a relatively low percentage, it is higher than many prior attempts to analyze community DNA from low biomass crustal samples (10, 11, 13, 27, 29). By comparison, a recent study described obtaining sufficient DNA for sequencing (after whole-genome amplification) from bulk nucleic acid extraction from >20 g of low biomass crustal samples with similar cell densities (30).

**TABLE 1:** Sample lithology and cell counts^*a*^

Sample^*b*^	Lithology	Cell density (cells/cm^3^)	PCR	Seq.	PFC INT	PFC EXT
**68B-1R1-1.44**	**Rubble: Metagabbro, with talc-amph overprinting**	**158**	**+**	**+**	**9 × 10^4^**	**ADL**
68B-3R1-3.77	Metasomatized serpentinite (top 10 to 13 cm, possibly gabbroic domains)	13	+	−	1 × 10^5^	1 × 10^5^
68B-3R1-3.80	Serpentinized harzburgite	61	+	+	5 × 10^3^	2 × 10^5^
68B-7R1-7.73	Mixed rubble: serpentinized harzburgite, talc-amph-chlorite schist	BMQL (4.4)	+	+	1 × 10^5^	3 × 10^5^
68B-8R1-9.02	Rubble: serpentinized harzburgite, talc-amph-chlorite schist	BMQL (4.4)	−	n.a.	1 × 10^4^	4 × 10^5^

69A-4R1-5.41	Foraminiferal carbonate sand	16,000	+	+	BDL	BDL
**69A-4R1-5.91**	**Foraminiferal carbonate sand with basalt fragments**	**5,010**	**+**	**+**	**BDL**	**2 × 10^2^**
69A-5R1-8.17	Metadolerite with carbonate veins	9	n.c.	n.a.	BDL	BDL
69A-7R1-11.05	Metadolerite rubble	5–16	n.c.	n.a.	2 × 10^2^	BDL
**69A-9R2-14.61**	**Serpentinized dunite with carbonate veins**	**10–24**	**+**	**+**	**3 × 10^2^**	**BDL-2 × 10^2^**

70C-1R1-0.59	Carbonate-hosted basalt breccia	29	−	n.a.	6 × 10^2^	3 × 10^4^
**70C-3R1-3.55**	**Carbonate-hosted basalt breccia**	**88**	**+**	**+**	**1 × 10^5^**	**1 × 10^6^**

71A-1R2-0.58	Serpentinized dunite, oxidized and with carbonate veins	405	−	n.a	BDL	7 × 10^4^

71B-2R1-2.33	Metagabbro (chloride-rich)	38	n.c.	n.a.	BDL	BDL

71C-2R1-3.51	Serpentinized harzburgite	n.d.	−	n.a.	BDL	1 × 10^5^
71C-3R1-5.1	Serpentinized harzburgite	n.d.	−	n.a	BDL	6 × 10^4^
71C-6R1-10.25	Metadolerite	n.d.	n.c.	n.a.	BDL	2 × 10^4^

72A-1R1-0.53	Rubble: basalt-breccia; carbonate matrix	18–499	n.c	n.a	BDL	BDL
						
72B-1R1-0.62	Rubble: basalt-breccia; carbonate matrix	241	n.c	n.a	1	1 × 10^1^
72B-3R1-3.78	Rubble of metabasalt, metagabbro, and serpentinized harzburgite	n.d.	n.c.	n.a	1 × 10^0^	BDL
72B-5R1-6.50	Talc-amphibole-chlorite schist	13	n.c.	n.a	1 × 10^0^	6 × 10^0^
72B-7R1-10.08	Metagabbro	4	n.c.	n.a	BDL	n.d.

74A-1R1-0.1	Carbonate sand (highly disturbed during core recovery)	22,400	−	n.a	n.a.	BDL
74A-1R1-0.5	Carbonate sand (highly disturbed during core recovery)	4,430	+	+	n.a.	6 × 10^2^

75B-2R1-2.95	Rubble: metadolerite	148	−	n.a.	2 × 10^3^	n.d.

76B-3R1-4.46	Metadolerite	29	−	n.a.	1 × 10^3^	BDL
76B-5R1-7.08	Serpentinized harzburgite with talc-amph-chlorite alteration	BMQL (4.8)	−	n.a.	BDL	3 × 10^1^
76B-7R1-10.76	Serpentinized harzburgite	BMQL (4.4)	n.c.	n.a.	BDL	2 × 10^3^
76B-9R1-13.21	Serpentinized harzburgite with talc-amph-chlorite alteration and carbonate veins	44	n.c.	n.a.	1 × 10^2^	8 × 10^1^

a Overview of samples used in this study, including sample depth, lithology, interior cell concentrations (from Früh-Green et al. [14]), whether PCR screens of MDA products after bulk cell sorting were positive (+) or negative (−) (PCR column), whether Illumina amplicon sequencing (Seq.) of MDA product was successful, and the concentration of PFC tracer in parallel samples taken from the interior (INT) or exterior (EXT) of the core on the ship, in picograms of perfluorocyclohexane per cm^3^ (from Orcutt et al. [66]). Samples used for metagenome and single-cell genome sequencing are highlighted in bold type. Abbreviations: ADL, above detection limit; BDL, below detection limit; BMQL, below minimum quantification limit; n.a., not applicable; n.c., no cells identified in FACS analysis; n.d., not determined.

b Samples are named as follows: hole-core section-top depth (meters below seafloor [mbsf]).

In addition to amplicon sequencing of the eight samples, four metagenomes were created and assembled together from four different samples: one sediment sample (357-69A-4R1-5.41 [sample naming method explained in Table 1, footnote *b*]) and three crustal samples (357-68B-1R1-1.44 mbsf, 357-69A-9R2-14.61 mbsf, and 357-70C-3R1-3.55 mbsf), resulting in 28.5 Mbp. The construction of 33 metagenome-assembled genomes (MAGs) from these metagenomes ranged in estimated completeness from <0.1% to 68% based on the presence of universal single copy genes (see Data Set S1 in the supplemental material). Finally, one sediment-basement interface sample (357-69A-4R1-5.41 mbsf) was sorted and low coverage sequencing was carried out, resulting in 227 single-cell amplified genomes (SAGs) ranging in estimated completeness from 0 to 29% (average, 6%; Data Set S1).

10.1128/mBio.00490-21.2 DATA SET S1 Excel spreadsheet of data. Download Data Set S1, XLSX file, 0.2 MB. Copyright © 2021 Goordial et al. 2021 Goordial et al. https://creativecommons.org/licenses/by/4.0/ This content is distributed under the terms of the Creative Commons Attribution 4.0 International license.

**FIG 4:**
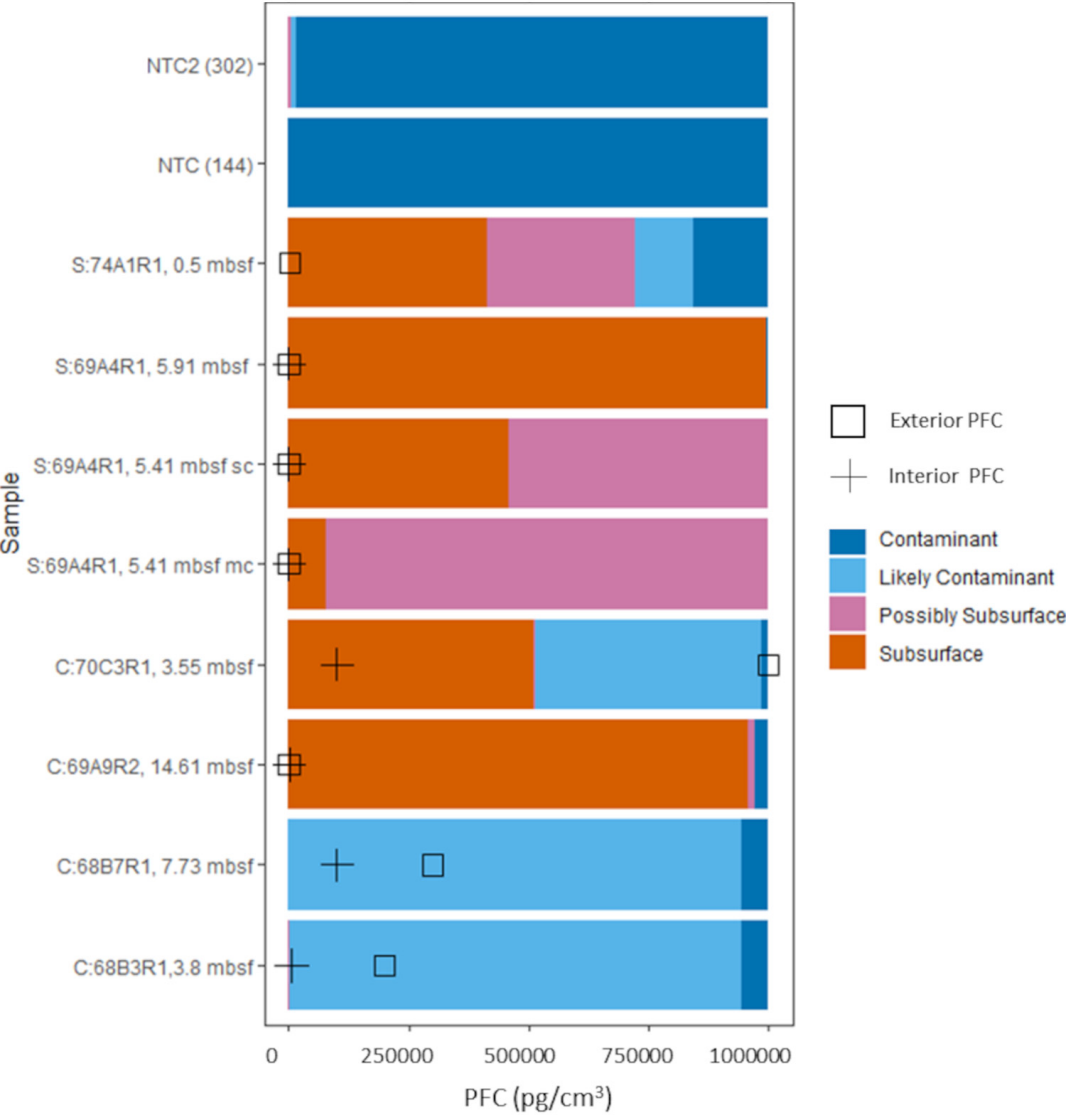
Relative abundance of 16S rRNA sequence origin categories in Atlantis Massif shallow subsurface crustal and sediment samples and no-template controls (NTC, top *x* axis) compared with perfluorocarbon (PFC) tracer concentrations (bottom *x* axis). Sequence categories were defined as sequences originating from the subsurface, possibly subsurface, likely contamination, and contamination, as defined in the text and the supplemental material. The concentration of PFC tracer was measured in parallel samples from both the interior (circles) or exterior (black squares) of the core as described in Orcutt et al. (66).

Amplicon sequencing negative controls yielded a total of 147 sequences, dominated by *Halomonas* (110 sequences [seq]) and *Shewanella* (21 seq). As the numbers of sequenced reads in the negative control were very small in relation to sample data sets (see Table S1 in the supplemental material), there was a possibility of falsely removing sequences due to contamination of negative controls from sample carryover (31). To understand the potential for sequenced taxa to represent contamination from molecular analyses, the drilling process, or the surface environment, we compared the 16S rRNA gene amplicon data in the present study to those obtained by Motamedi et al. (27) that included 14 drilling fluid samples and 212 water column and serpentinite samples from IODP Expedition 357. To further explore whether the sequences in the subsurface-origin categories reflected that habitat origin, representative amplicon sequence variant (ASV) sequences from abundant taxa (taxa with >5,000 reads) were searched against the GenBank database to determine percent similarity to existing sequences and the environments of the closest matches. Finally, we carried out phylogenetic analyses on selected MAGs and SAGs using concatenated conserved genes in order to assess similarity to taxonomic relatives (and the environments these sequences were derived from; see Fig. S4a-c in the supplemental material). These methods are described in detail in Text S1 in the supplemental material. We used these data to classify sequences as originating from “Subsurface, Possibly Subsurface, Likely Contaminant, and Contaminant” (Fig. 4). Samples with a higher percentage of amplicon reads identified as subsurface in origin generally corresponded with samples with low drilling fluid tracer (i.e., perfluorocarbon [PFC]) concentrations (Table 1). Suspected contaminant sequences were removed from all subsequent amplicon analyses, resulting in the removal of 20% of sequence data from seven of the eight samples and the complete exclusion of one crustal sample amplicon data set (i.e., 357-68B-3R1-7.73 mbsf), which was composed entirely of suspected contaminants. While three samples used for metagenomic sequencing did have detectable PFC contamination tracer concentrations, the sequence grouping categorization described above indicates that the 16S rRNA gene sequences from these samples are of subsurface origin. Thus, we feel confident in describing the bulk metabolic functional potential interpretation from these data sets.

10.1128/mBio.00490-21.1 TEXT S1 Supplemental methods. Download Text S1, DOCX file, 0.03 MB. Copyright © 2021 Goordial et al. 2021 Goordial et al. https://creativecommons.org/licenses/by/4.0/ This content is distributed under the terms of the Creative Commons Attribution 4.0 International license.

10.1128/mBio.00490-21.6 FIG S4 Phylogenomic placement of MAGs and SAGS supports subsurface origin of *Acidobacteria* (A), *Chloroflexi* (B), Dadabacteria (C) taxa. MAGs from this study are labeled as “bins.” SAGs generated in this study are labeled with SAG ID with prefix AH-259. Other related genomes are labelled with GenBank or IMG accession number and a description of the environment that the isolate or metagenomic DNA was obtained from. MAGs with less than five identified marker genes for concatenation are denoted with an asterisk. Download FIG S4, DOCX file, 0.5 MB. Copyright © 2021 Goordial et al. 2021 Goordial et al. https://creativecommons.org/licenses/by/4.0/ This content is distributed under the terms of the Creative Commons Attribution 4.0 International license.

10.1128/mBio.00490-21.8 TABLE S1 Numbers of OTUs in amplicon surveys. Summary of Illumina amplicon sequencing of bulk cell sorted samples from IODP Expedition 357, indicating total number of paired-end (PE) sequence reads and number of estimated operational taxonomic units (OTUs), calculated at 97% sequence similarity or higher, without subsampling to smallest sample size). One sample was bulk cell sorted into two tubes, and the MDA reaction was performed with two different REPLI-g kits (minikit and single-cell kit). One negative control from the MDA reactions was also included to assess for possible sequence contamination. Download Table S1, DOCX file, 0.01 MB. Copyright © 2021 Goordial et al. 2021 Goordial et al. https://creativecommons.org/licenses/by/4.0/ This content is distributed under the terms of the Creative Commons Attribution 4.0 International license.

**FIG 5:**
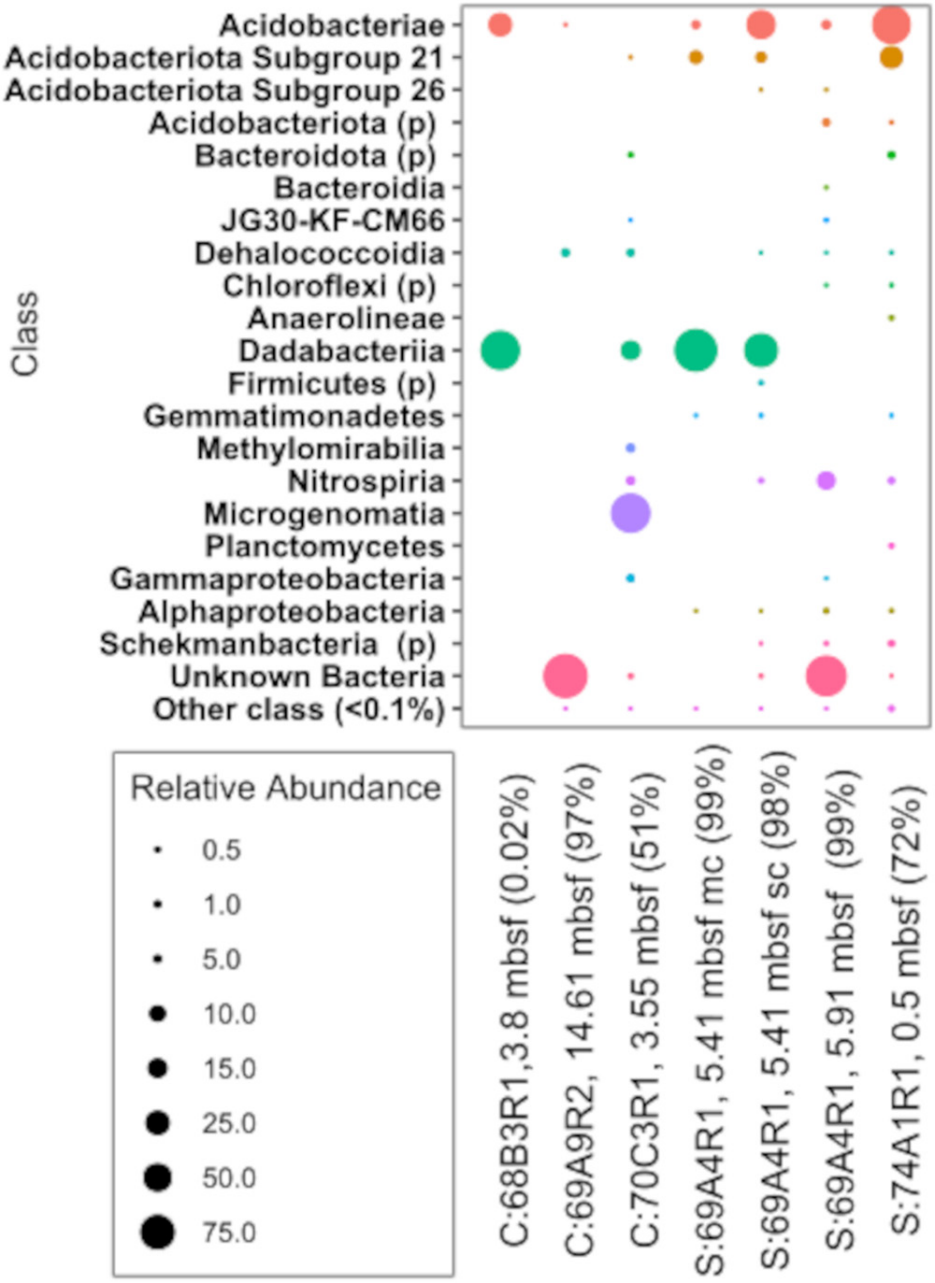
Relative abundance of amplicon 16S rRNA gene (v4v5) sequences in Subsurface/Possibly Subsurface category taxa from bulk sorts of ∼1,000 cells from (at family level) crust (C) and sediment (S) samples of the shallow subsurface of Atlantis Massif. Families which had less than 0.1% relative abundance across all samples were grouped into the “other family” category. Sediment sample 69A4R1-5.41 mbsf was processed and sequenced with two whole-genome kits: the REPLI-g single-cell kit (sc) and the REPLI-g minikit (mc). ASVs that were considered to be contaminants or likely contaminants are not shown. (p) denotes where taxa could not be resolved beyond the phylum level.

Based on 16S rRNA gene amplicon sequences that passed contamination screening, the bacterial community composition of the Atlantis Massif shallow subsurface sediment and crust samples was heterogeneous and low in diversity (Fig. 5). Caution should be taken when evaluating amplicon-based relative diversity levels between samples, as the cell extraction step did not recover all cells (Fig. S1), the cell concentration steps may not have recovered all cell types equally, and the whole-genome amplification applied to concentrated bulk cell sort DNA is likely to overamplify some groups (32). However, presence-absence comparisons are useful. For example, most abundant taxa were present in both sediment and crustal libraries (Fig. 5), such as *Acidobacteria*, “*Candidatus* Dadabacteria,” *Dehalococcoidia*/*Chloroflexi*, and taxa classified as unknown bacteria.

10.1128/mBio.00490-21.3 FIG S1 Evaluation of cell separation methodology. (A) Cell quantification in test rock samples via flow cytometry in samples after “gentle” cell extraction using salt buffers, a “harsh” cell extraction using a Nycodenz density gradient solution (as described by Morono et al. [Y. Morono, T. Terada, J. Kallmeyer, and Inagaki F, Environ Microbiol 15:2841-2849, 2013, https://doi.org/10.1111/1462-2920.12153]), and a harsh cell extraction on samples after the gentle extraction had been carried out. (B) Effect of different cell separation methods on flow cytometry (FC) sorting and DNA amplification. In the flow cytometry assessment column, more “+” symbols indicate qualitatively more cell-like particles observed during FACS. For the DNA concentration assessment column, symbols indicate relative amount of DNA quantified in sample after whole-genome amplification as follows: +++, >600 ng/μl; +, 10 to 100 ng/μl; −, <5 ng/μl. Download FIG S1, DOCX file, 0.1 MB. Copyright © 2021 Goordial et al. 2021 Goordial et al. https://creativecommons.org/licenses/by/4.0/ This content is distributed under the terms of the Creative Commons Attribution 4.0 International license.

Within the *Acidobacteria,* there were several clades observed. In addition, further phylogenetic analysis of the unknown bacteria 16S rRNA gene operational taxonomic units (OTUs) revealed that 30 of 39 of these OTUs formed a clade most closely related to *Acidobacteriae* subgroup 2 sequences from other terrestrial subsurface environments (33, 34) (Fig. S3). The 16S rRNA gene similarity of this clade of Atlantis Massif OTUs with other *Acidobacteria* ranges from 82.4 to 85.8%, which passes the threshold for suggesting that these organisms may be candidates of a novel class or phylum-level lineage (35). These OTUs were distinct from *Acidobacteriae* subgroup 2 ASVs identified in amplicon analysis (Fig. 5), which formed a separate related clade. Phylogenetic trees of concatenated conserved genes in assembled MAGs similarly revealed clades of *Acidobacteria* (Fig. S4A). We identified one clade most similar to genomes from subsurface metal-contaminated sediments (36) and clades related to deep seawater. By comparing sequences generated in this study at the ASV level with those from sequenced seawater samples obtained during the drilling expedition, we could identify only one acidobacterial ASV (subgroup 2) that could be a potential contaminant from seawater. However, this ASV was also identified in higher abundance in serpentinites that seawater in the Motamedi study and was considered to be likely indigenous (27). The remaining *Acidobacteria* subgroup 2 sequences unique to this study were all found to be most similar to those from other terrestrial and marine subsurface environments (Fig. S3). Taken together, these results suggest these *Acidobacteria* are true subsurface inhabitants.

10.1128/mBio.00490-21.5 FIG S3 Phylogenetic placement of unclassified bacterial OTUs with the *Acidobacteria* phyla from 16S rRNA gene amplicon sequencing of Atlantis Massif sorted cells. Bootstrap values supporting branching order of phylogenetic tree are displayed as circular symbols at nodes, with no symbols on nodes that have a bootstrap value of less than 50. Collapsed branch consists of proteobacterial sequences as an outgroup. The inner ring provides the name of the OTU or SAG from this study (see Data Set S1) or the NCBI GenBank accession number for closest sequence relatives from published studies, with color denoting *Acidobacteria* subgroup classification as indicated in the legend. The outer ring indicates the environment type for the closest environmental sequences or the presence (filled)/absence (unfilled) of the OTU in each sample from this study: NTC, no template control; S1, 74A-1R1-0.5mbsf; S2, 69A-4R1-5.91mbsf; S3, 69A-4R1-5.41mbsf (mini MDA kit); S4, 69A-4R1-5.41mbsf (single-cell MDA kit); C1, 70C-3R1-3.55mbsf; C2, 69A-9R2-14.61mbsf; C3, 68B-7R1-7.73mbsf; C4, 68B-3R1-3.8mbsf. Download FIG S3, DOCX file, 1.1 MB. Copyright © 2021 Goordial et al. 2021 Goordial et al. https://creativecommons.org/licenses/by/4.0/ This content is distributed under the terms of the Creative Commons Attribution 4.0 International license.

*Dehalococcoides* (*Chloroflexi*) were found in both crust and sediment amplicon and metagenome samples. MAGs and SAGs formed separate clades with most similarity to other sediments, which included Arctic Ocean Mid-Ocean Ridge sediment and metal contaminated aquifer sediments (Fig. S4B). “*Ca*. Dadabacteria” sequences comprised a large proportion of reads from two crust samples and a foraminiferal carbonate sand and were most closely related to environmental sequences from hot springs, hydrothermal vents, and the terrestrial subsurface (Fig. S4C). Microgenomatia (“*Candidatus* Patescibacteria”), which are widely observed in subsurface environments (37, 38), were abundant only in one crustal sample (Fig. 5). Methylomirabilia, an organism associated with anaerobic oxidation of methane (39) was identified in the 16S rRNA amplicon sequences of one crustal sample (357-70C-3R1-3.55 mbsf).

The microbial genera identified in the shallow subsurface of the Atlantis Massif by amplicon sequencing were distinct from those described previously from LCHF fluids and chimneys, which are dominated by methane-oxidizing *Methylomonas*, sulfate-reducing *Thiomicrospira*, and the archaeal methanogens and methanotrophs *Methanosarcinales* and ANME-1 (20, 21, 23). This indicates that endolithic communities in the shallow subsurface of the Atlantis Massif are distinct from those in the actively venting LCHF system. While the shallow Atlantis Massif cores have clear evidence of serpentinization in the highly altered lithologies recovered, it is likely that the LCHF system is sourced from deeper fluids than would have been accessed during the shallow drilling of IODP Expedition 357 (14, 24). Previous work in shallow gabbroic rock samples from the Atlantis Massif suggested that the microbiota are composed of widespread and ubiquitous generalists (29), with similar taxa also reported in deep gabbros from the Atlantis Bank in the Indian Ocean (30). The new results from Atlantis Massif contrast to the results of the prior studies, which may reflect the different approaches used and how potential contamination was tracked. Moreover, recent studies have documented differences between rock-hosted biofilm microbial communities and those within the fluids circulating through subsurface crust (40), which is consistent with our results. Regardless, the results of phylogenomic analysis of the shallow subsurface Atlantis Massif samples (Fig. S4a to e, Fig. S3, and Table S2) support a subsurface origin for these groups.

10.1128/mBio.00490-21.9 TABLE S2 Environment of closest blast match of abundant (>5,000 reads) amplicon contaminants. Download Table S2, DOCX file, 0.01 MB. Copyright © 2021 Goordial et al. 2021 Goordial et al. https://creativecommons.org/licenses/by/4.0/ This content is distributed under the terms of the Creative Commons Attribution 4.0 International license.

No archaeal sequences were observed in amplicon libraries, though one MAG classified as a *Nanoarchaeota* (51% complete) and two SAGs classified as *Crenarchaeota* (18 to 23% complete) were obtained from sample 357-69A-4R1-5.41 mbsf. The pattern of low to no archaeal reads found in the genomic data sets are similar to 16S rRNA gene amplicon and metagenomic surveys in fractures from the deep terrestrial subsurface (41) and in lower gabbroic crust from the Southwest Indian Ridge (30) but are in contrast to abundant *Archaea* found in hot anoxic crustal fluids at the Juan de Fuca Ridge flank (42, 43) and to the *Archaea*-dominated (*Methanosarcinales*) carbonate chimneys of the LCHF (20, 21). If we assume that there is no preferential bias against archaeal cells with the cell extraction approaches used in this study, our results again point to a rock-associated community in the Atlantis Massif shallow subsurface distinct from communities in the high pH fluids of LCHF.

### Different approaches yield different subsurface community compositions.

**FIG 6:**
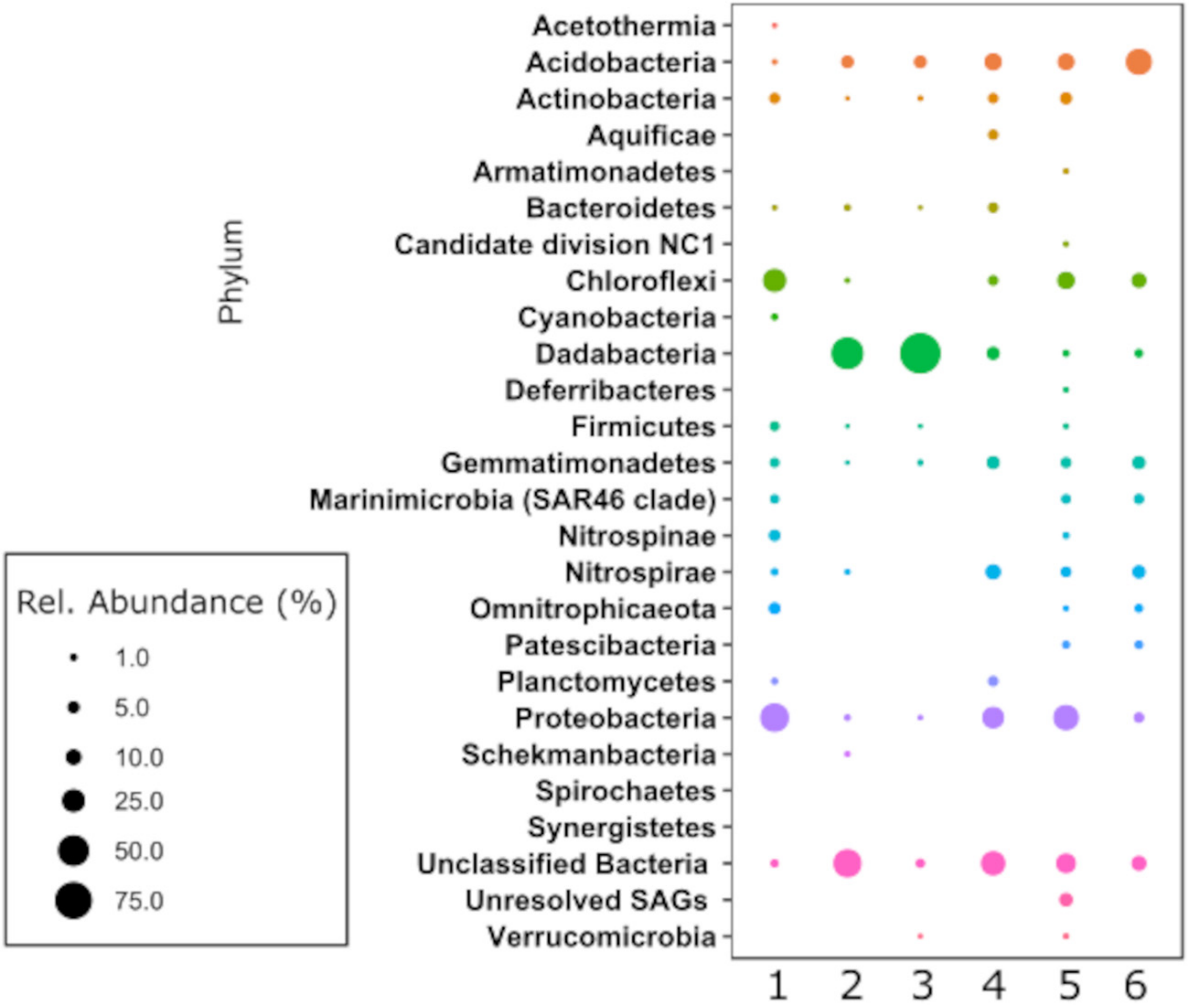
Comparison of -omics approaches employed in the present study. Relative abundance (as a percentage) of bacterial sequences (phylum level) for different DNA extracts generated by different approaches from cells concentrated and sorted from one subsurface sample (from sediment sample 357-69A-4R1-5.41 mbsf). Lanes: 1, amplicon sequences from bulk extractions as in Motamedi et al. (27), total 44,443 sequences (seqs); 2 and 3, 16S rRNA gene V4 hypervariable region amplicon sequences from ∼1,000 cells sorted together using two different MDA kits, the Repli-G single-cell kit (total 49,920 seqs) (lane 2) and Repli-G minikit (total 59,649 seqs) (lane 3); lane 4, bacterial metagenome-assembled genomes (MAGs) from metagenomic DNA sequences generated from ∼1,000 cells sorted together, total of 20 MAGs; lanes 5 and 6, bacterial single-cell amplified genomes (SAGs) from cells sorted individually, with taxonomic identity indicated by either from checkM ID of SAG DNA after low-coverage (LoCoS) sequencing (lane 5) (total of 227 SAGS) or from Sanger sequencing of the 16S rRNA gene (lane 6) (total of 93 seqs).

One Atlantis Massif subsurface sample—sample 357-69A-4R1-5.41 mbsf from the sediment-basement interface—was sequenced with amplicon (two replicates with different multiple displacement amplification [MDA] kits), metagenomic and single-cell genomic approaches, resulting in an amplicon library with 199 to 386 OTUs, 20 metagenome-assembled genomes, and a plate of 227 single-cell amplified genomes. In addition, we compared these data with amplicon sequencing results obtained from a bulk DNA extraction protocol (27). The microbial diversity suggested by each of the sequencing approaches differed substantially (Fig. 6), highlighting the influence that amplification bias has on community structure analysis from low biomass samples. For example, cell sort amplicon data indicated the dominance of candidate phylum Dadabacteria (formally SBR1093); however, only one MAG and one SAG belonging to Dadabacteria was identified in the metagenome and single-cell genome data, respectively. The highest diversity of phyla observed was from the single-cell genomics data set of 227 SAGs. Within this plate, the best taxonomic classifications resulted from Sanger sequencing of the 16S rRNA gene (which was successful for 94 of the 227 SAGs), as the partial genomes obtained by low-coverage genome sequencing often did not contain sufficient marker genes for identification. The SAG-based community structure was most similar to that generated from bulk DNA extraction and sequencing techniques in terms of relative abundance of phyla (27). Despite the fact that the “gentle” extraction technique did not quantitatively recover all cells, our results indicate that the relative community structure was still maintained and not biased by the cell extraction technique. Thus, SAG-level microbial community structure analysis of low biomass samples, even with low-coverage genome sequencing, is likely a more accurate representation of relative diversity compared to the other cell sorting-based methods that pool cells and amplify DNA, reflecting the bias introduced from multiple displacement amplification and primer bias in the bulk cell sorts. We highlight here that the SAG-based approach required only a few grams of sample material, whereas bulk DNA extraction techniques in other studies required concentration of DNA from several dozens of grams of material (27, 30).

### Microbial functional potential in the Atlantis Massif subsurface.

**FIG 7:**
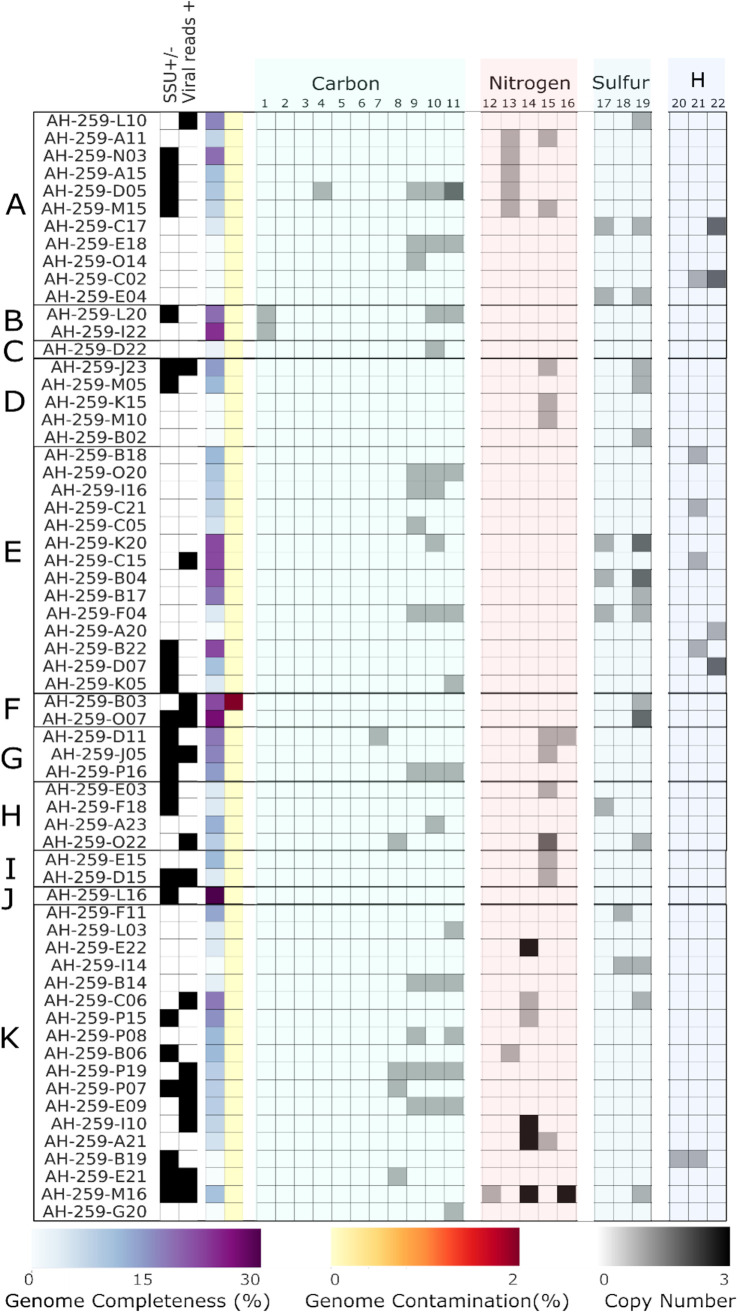
Metabolic functional potential of selected SAGs from sediment sample 357-69A-4R1-5.41 mbsf. Organization of *y* axis: bacterial or archaeal phyla of each SAG: *Acidobacteria* (A), *Crenarchaeota* (B), *Armatimonadetes* (C), unclassified bacteria (D), *Chloroflexi* (E), “Ca. Dadabacteria” (F), *Gemmatimonadales* (G), “*Ca*. Marinimicrobia” (H), *Nitrospira* (I), “Ca. Patescibacteria” (J), and *Proteobacteria* (K). Organization of *x*-axis columns: presence (filled)/absence (unfilled) of ribosomal small subunit (SSU) and/or viral reads in SAG, estimated completeness of SAG based on single copy marker genes (scaled from 0 to 30% per legend), estimated percentage of contamination of genome from checkM (scaled from 0 to 2% per legend), and copy number of metabolic function genes (scaled from 0 to 3 copies per legend) grouped as RuBisCO (1), phosphoribulokinase (2), carbon monoxide dehydrogenase (CoSH)/acetyl-CoA synthase (ACS) (3), acetyl-CoA synthase (4), 4-hydroxybutyryl-CoA dehydratase (5), methane monooxygenase (6), isocitrate lyase (7), malate synthase (8), CODH large chain (9), CODH medium chain (10), CODH small chain (11), ammonia monooxygenase (12), periplasmic nitrate reductase (13), respiratory nitrate reductase (14), nitrite reductase (15), nitric oxide reductase (16), adenylylsulfate reductase (17), phosphoadenosine phosphosulfate reductase (18), sulfite reductase (19), periplasmic [NiFe] hydrogenase (20), periplasmic [NiFeSe] hydrogenase (21), and NADP-reducing hydrogenase [FeFe] (22).

**FIG 8:**
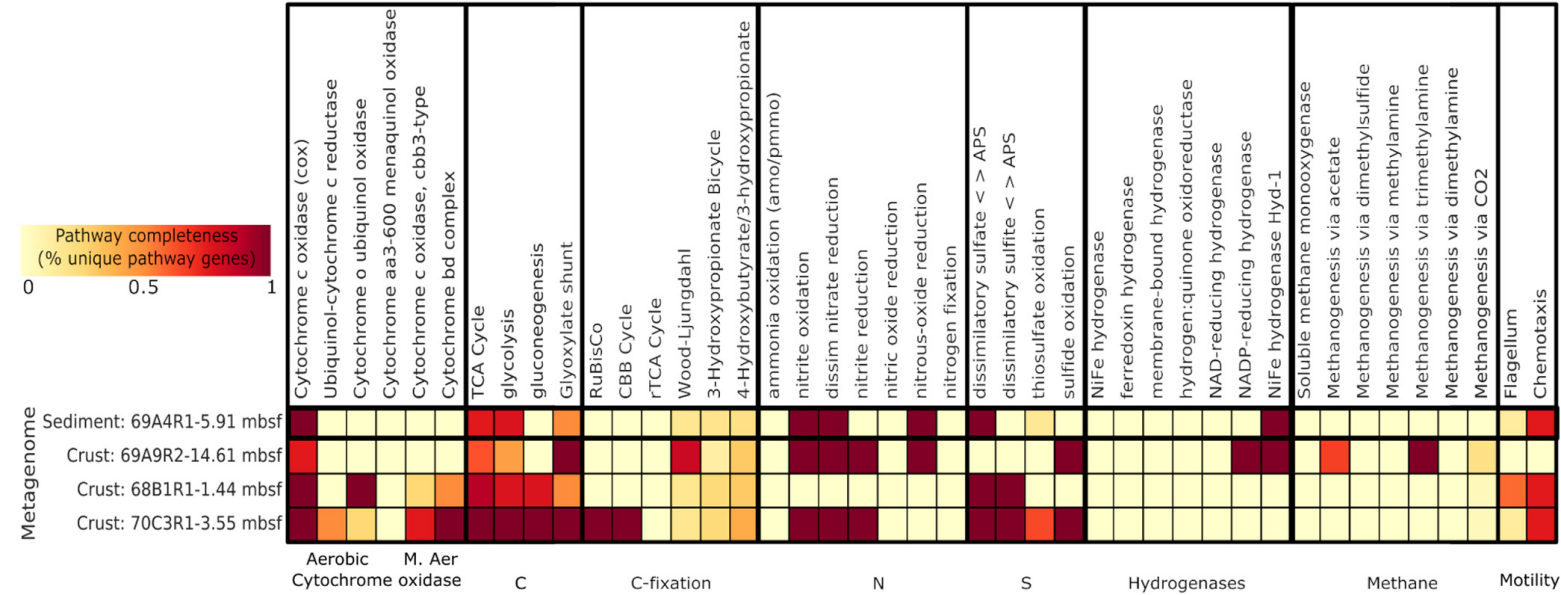
Metabolic potential in subsurface sediment and crustal microbial communities from Atlantis Massif IODP Expedition 357 samples based on metagenomes constructed from bulk cell sorts. Pathway completeness is a measure of the percentage of genes unique to that pathway as in Graham et al. (76). M. Aer oxidase, microaerobic cytochrome oxidase.

The four metagenomes and 227 low-coverage SAGs were screened for essential genes in known respiratory and carbon fixation pathways, methane metabolism, and sulfur and nitrogen cycling (Fig. 7 and 8; see also Data Set S1 in the supplemental material). Overall, heterotrophic metabolisms in the Atlantis Massif shallow subsurface appears common, as the tricarboxylic acid (TCA) cycle was observed in all metagenomes, and it was prevalent in low-coverage SAGs and MAGs, as were genes associated with glycolysis/gluconeogenesis. In contrast, key genes for autotrophic carbon fixation pathways were rare. For example, essential genes for the reductive TCA cycle (ATP citrate lyase, 2-oxoglutarate:ferredoxin oxidoreductase) were not found in any data set. Some genes related to the 3-hydroxypropionate bicycle (3-HP bicycle) and the 3-hydroxypropionate/4-hydrocybutyrate cycle were detected, but only one acetyl coenzyme A (acetyl-CoA)/propionyl-CoA carboxylase (key gene of 3-HP bicycle) was detected in all four metagenomic libraries. Putative RuBisCO genes and a nearly complete Calvin-Benson-Bassham (CBB) cycle were found in crustal metagenome 357-70C-3R1-3.55 mbsf; however, it could not be associated with a MAG (Fig. S5). In the SAGs, the potential for carbon fixation via the CBB was identified in *Crenarchaeota* (AH-259-L20 and AH-259-I22) based on the presence of RuBisCO (Fig. 7). The presence of CBB and reductive TCA (rTCA) autotrophic pathway genes have previously been associated with ultramafic-hosted vent fluids and chimneys from the Lost City and Logatchev hydrothermal fields on the Mid-Atlantic Ridge (19, 44). The Wood-Ljungdahl (WL) pathway for anaerobic autotrophy is thought to potentially have emerged in serpentinizing systems, such as the Atlantis Massif, and may be one of the oldest metabolisms on Earth (45, 46). One *Dehalococcoidetes* MAG (MAG 22; 51% complete, 0.99% estimated genome contamination) encoded a nearly complete WL pathway, missing two enzymes in the pathway (methylenetetrahydrofolate reductase, *metF*, and 5-methyltetrahydrofolate methyltransferase, *ascE*). This MAG contained the only anaerobic carbon monoxide dehydrogenase (CODH)/acetyl-CoA synthase (ACS) complex found in all MAGs and SAGs, as well as key genes for nitrite reduction, sulfite reduction, and hydrogenases (Ni-Fe-Se/Fe-Fe; Fig. S5). An incomplete WL pathway occurs in Dehalococcoides mccartyi strain 195, which also lacks *metF*, and nonetheless can incorporate both formate and CO into biomass via an alternative pathway for methyl-tetrahydrofolate production (47). Reads for *Dehalococcoidetes* MAG22 were recruited entirely from the crustal metagenome from sample 357-69A-9R2-14.61 mbsf, a serpentinized dunite that was the deepest sample obtained from the site closest to the LCHF (Fig. 1).

10.1128/mBio.00490-21.7 FIG S5 Expanded view of the metabolic potential of individual metagenome-assembled genomes (MAGs). MAGs grouped on the *y* axis by taxonomy. Columns along *x* axis: estimated completeness of MAG based on single copy marker genes (scaled from 0 to 100% per legend), estimated percentage of contamination of genome from checkM (scaled from 0 to 16% per legend), and copy number of metabolic function genes (scaled from 0 to 5 copies per legend) grouped as C-cycling pathways RuBisCO (1), phosphoribulokinase (2), carbon monoxide dehydrogenase (CoSH)/acetyl-CoA synthase (ACS) (3), ACS (4), 4-hydroxybutyryl-CoA dehydratase (5), methane monooxygenase (6), isocitrate lyase (7), CODH large chain (8), CODH medium chain (9), CODH small chain (10), nitrogen cycling pathways (11), ammonia monooxygenase (12), periplasmic nitrate reductase (13), respiratory nitrate reductase (14), nitrite reductase, nitric oxide reductase (15), sulfur-cycling pathways adenylylsulfate reductase (16), phosphoadenosine phosphosulfate reductase (17), sulfite reductase and hydrogenases (18), periplasmic [NiFe] hydrogenase (19), periplasmic [NiFeSe] hydrogenase (20), and NADP-reducing hydrogenase [FeFe] (21). Download FIG S5, DOCX file, 0.2 MB. Copyright © 2021 Goordial et al. 2021 Goordial et al. https://creativecommons.org/licenses/by/4.0/ This content is distributed under the terms of the Creative Commons Attribution 4.0 International license.

While there was limited evidence for autotrophy in samples in this study, aerobic carbon monoxide dehydrogenases were widespread in diverse phyla in the SAG and MAG data sets, suggesting CO metabolism in the Atlantis Massif shallow subsurface may be important for energy conservation. CO assimilation and metabolism are thought to potentially be important in serpentinizing systems, where CO_2_ is limited due to alkaline conditions (16). Aerobic carbon monoxide dehydrogenase subunits were found in five MAGs (from the *Acidobacteria*, *Chloroflexi*, and *Proteobacteria*) and in 21 diverse SAGs (11% of SAGS ranging from 0 to 21% completeness; Fig. 7; see also Fig. S5 and Data Set S1). The crenarchaeal SAGs (AH-259-L20 and AH-259-I22) with aerobic CO dehydrogenases also contained RuBisCO coding sequences (CDSs) associated with the CBB (Fig. 7). Aerobic CO-oxidizing microorganisms are widespread in both marine (48) and terrestrial settings (49). However, previous metagenomic surveys of Lost City chimneys indicate this gene was rare, with only one shotgun read recovered (50). CO oxidation can be used to conserve energy supporting aerobic heterotrophic organisms in oligotrophic or nutrient-stressed conditions (49, 51), while some aerobic carboxydotrophs can use the energy conserved to support carbon fixation through the CBB cycle (49, 52). The importance of energy and carbon conservation in the Atlantis Massif subsurface is also suggested by the presence of isocitrate lyase and malate synthase, key genes in the glyoxylate shunt pathway (Fig. 7 and 8 and Data Set S1). Six of the MAGs and nine SAGs had at least one of these key genes. This pathway enables the CO_2_ releasing steps of the TCA cycle to be bypassed, conserving carbon in an oligotrophic environment. The pathway enables microorganisms to use acetate as the sole carbon source (53). Acetate has been observed in micromolar concentrations in LCHF fluids (54).

While autotrophic metabolisms common in LCHF fluids were absent in the metagenomic data sets, the ability for formate utilization was present in our data (Data Set S1), which is consistent with prior observations of formate-dependent metabolisms at the LCHF (17, 23). Formate is associated with serpentinizing systems and has been detected in deeply sourced Atlantis Massif hydrothermal vent fluids (16, 54, 55). Several types of formate dehydrogenases and associated transporters (including oxalate:formate antiporters) were found throughout all data sets. One acidobacterial MAG (MAG 31) had multiple putative types: the alpha subunit of formate dehydrogenase (FDH), formate dehydrogenase N (FDN), and formate dehydrogenase O (FDO). FDO allows the use of formate as an electron donor with oxygen as a terminal electron acceptor, while FDN is induced by nitrate during anaerobic growth (56). Seven SAGs contained formate dehydrogenase H (FDH), part of the fermentative hydrogenlyase complex (57) (six *Alphaproteobacteria* and an *Acidobacteria* subgroup 21). Formate dehydrogenase, as well as formate-tetrahydrofolate ligase, a key enzyme that acts upon formate in the WL pathway, were identified in *Dehalococcoidetes* MAG 22. As autotrophic pathways are largely absent in our data, it is unlikely that formate plays a role in anabolic processes in most organisms. Rather, available formate may be used as an electron donor for energy conservation in the subsurface, and/or it might also be assimilated by heterotrophs.

No essential genes for methane metabolisms, such as methane monooxygenase (*mmoX*, *pmoA*) or methyl coenzyme M reductase associated with methane oxidation/methanogenesis, were found in any MAGs or SAGs generated in this study, suggesting that methane cycling is not a dominant metabolism in the shallow serpentinite subsurface of the Atlantis Massif. This is in contrast to methane cycling observed to support LCHF venting fluids and chimney microbiota (17, 21, 22, 58) and the observation of elevated methane in Atlantis Massif samples during drilling (2 to 48 nM in samples compared to below detection limit [0.7 nM] in bottom water) (14), though consistent with cultivation attempts in parallel samples, which did not identify any methanogens (59). Acetyl-CoA decarbonylase/synthase complex genes associated with methanogenesis via acetate (via the WL pathway), and trimethylamine methyltransferase associated with methane production via trimethylamine, were found in the deepest crustal metagenome from sample 357-69A-9R2-14.61 mbsf (Fig. 8). Alkanes produced in the Atlantis Massif subsurface and found in hydrothermal fluids were thought to be a potential C source in this environment (16). Alkane degradation genes associated with known aerobic and anaerobic pathways were rare, with only one propane and butane monoxygenase recovered from crustal metagenomes (Data Set S1). Thus, the use of alkanes as a major nutrient source for crustal microbiota in the shallow subsurface of Atlantis Massif is not supported by metagenomic evidence in our study.

Beyond carbon cycling potential, we also examined terminal electron acceptor pathways in the Atlantis Massif subsurface. Aerobic-type cytochrome oxidases were the most prevalent in all metagenomes (Fig. 8), with subunits for cytochrome *c* oxidase found in 22% of SAGs and 51% MAGs generated. Oxygen-sensitive cytochromes Cbb and Bd type were found in 4% of SAGs and 6% of the MAGs, and recruited from only crustal metagenomes (Fig. 8). Key genes associated with denitrification (nitrate reductase, nitrite reductase, nitric oxide reductase) were recovered, though respiratory nitrate reductase could not be binned to a MAG. Respiratory nitrate reductases were found in six proteobacterial SAGs (Data Set S1). The metabolic potential for ammonia oxidation or nitrogen fixation was not observed in the metagenomic sequences (Fig. 8). This is in contrast to the diversity of nitrogen fixation genes found in biofilms from carbonate chimneys at the LCHF (58) but similar to the observation of nitrogen cycling potential in deep gabbros of the Atlantis Bank (30). S-cycling genes as sulfate and sulfite reductases were found in all data sets (Fig. 7 and Fig. S5). Sulfate reduction is an important metabolism in LCHF vent fluids and likely in the subsurface, while chimney exteriors are associated more with sulfur oxidation (17). Potential metal-respiring and metal-associated CDSs were found in all metagenomes (Data Set S1) and indicate that detoxification and resistance to metals such as mercury and arsenic may additionally be important in the Atlantis Massif subsurface. Thus, overall, diverse aerobic and anaerobic metabolic strategies appear to be supported throughout the Atlantis Massif shallow subsurface.

Virus-like sequences were identified in 9 (of 33) MAGs and in 60 (of 227) SAGs, resulting in ∼26% of the partially complete genomes with evidence of past or current viral interactions (Fig. 7 and Data Set S1). The virus-like sequences discovered in this study may be coming from either active infections or prophages. As the genome completeness estimates were low for the MAGs and SAGs, cells with evidence of viral interactions may be higher than observed (60). Viral sequences were found associated with sediment SAGs across a diversity of phyla, including *Acidobacteria*, *Chloroflexi* (*Dehalococcoides*), “*Ca*. Dadabacteria,” *Gemmatimonadetes*, *Gammaproteobacteria* (*Halomonas*), Deltaproteobacteria (*Deferrisoma*), “*Candidatus* Marinimicrobia” (SAR406), *Nitrospirae*, *Nitrospinae*, and *Alphaproteobacteria*. Alphaproteobacterial SAGs classified as *Rhodospirillales* had the highest number of associated viral reads, representing 8 of the 39 SAGs with viral sequences in category 1 and category 2. Because multiple displacement amplification (MDA) amplifies only double-stranded DNA (dsDNA) and single-stranded DNA (ssDNA), RNA viruses were not detected in this study. For comparison, warm, anoxic crustal fluid flowing through the basaltic basement at the Juan de Fuca Ridge flank identified abundant viruses in the crustal fluids, with archaeal viruses the most common, and only 20 to 36% of viruses predicted to infect bacterial cells (61). This is the second documentation of the host phylogenetic affiliation of viruses in the crustal biome, and the first to include cells from rock as opposed to solely in the crustal fluids, though infected cells from fluids would have been captured in our cell extraction methods as well. These data in rocks and sediments add to growing evidence that viral processes extend to the deep biosphere (61), and may play an important role in evolution and ecological functioning, even in low biomass environments.

### Conclusion.

The marine crustal subsurface is an undersampled enigmatic habitat, yet a deep biosphere in lithosphere has consequences for global biogeochemical cycling. In addition to the logistical obstacles with obtaining samples suitable for microbial analysis, low cell numbers make molecular approaches that identify function and diversity inherently difficult. By using FACS to concentrate cells from grams of low biomass (10^1^ to 10^4^ cells g^−1^) subsurface crust and sediment samples to enable downstream ‘omic analyses, this study represents the first metagenomic and single-cell genomic analysis of ultramafic rock and sediment in a marine serpentinizing system. This approach revealed that microbial communities in the shallow subsurface of Atlantis Massif are predominantly heterotrophic. This supports the interpretation that, as biologically available CO_2_ is scarce due to low dissolved inorganic carbon (DIC) and high alkalinity, it is likely that heterotrophic organisms are foundational in serpentinizing systems (62). Such organisms would have to be poised to metabolize abiotically produced small organic molecules like formate and CH_4_ or would need access to circulating seawater carrying dissolved organic carbon sourced from elsewhere. Additionally, we identified a potential role for carbon monoxide oxidation as a supplementary energy source in the shallow subsurface. The insights generated by this new cell sorting-based approach increase our understanding of microbial metabolism and composition in low biomass oceanic crust environments. These results are also relevant to the field of astrobiology, as serpentinizing systems are of interest both for the origins of life on Earth and the habitability of other planetary bodies such as Enceladus, a moon of Saturn, or Mars, where serpentinization reactions are thought to have occurred (63–65).

## MATERIALS AND METHODS

### Sample collection.

Samples of ultramafic and mafic rocks and sediment (Table 1) from the southern wall of the Atlantis Massif were collected during IODP Expedition 357 (October-December 2015, RRS *James Cook*), as detailed elsewhere (14) (Fig. 1). The seabed drills were equipped with tracer delivery systems to allow for evaluation of the potential for contamination of the samples (66). For this study, approximately 5-cm^3^ subsamples of core were extracted by chisel from the whole round cores after flame sterilization of the exterior in a shipboard clean air system (KOACH model T-500-F; Koken, Ltd., Tokyo, Japan) to avoid potential airborne contaminants (e.g., dust). Sediment subsamples were collected via cut-end syringes inserted into the core interior. The subsamples were immediately mixed with a buffer (1× GlyTE [5% glycerol {final concentration} in 1× Tris-EDTA buffer {pH 8}]) in 15- or 50-ml centrifuge tubes and frozen at −80°C until shore-based analysis. Parallel subsamples from these same cores were collected for contamination tracer, cell density, and bulk community DNA extraction analysis, as presented elsewhere (14, 27, 66).

### Cell extraction, sorting, and DNA amplification from environmental samples.

The FACS cell concentration approach to enable downstream sequencing required cells to be extracted from the rock and sediment matrix into liquid buffer (Fig. 2). Following testing of various cell extraction protocols, we developed a “gentle” method for cell extraction that employed only physical disruption in salt buffers (described in Text S1 in the supplemental material). While this “gentle” method does not result in complete liberation of all cells from test rock samples, acids, detergent, and density gradient reagents commonly used for quantitative cell extraction (“harsh” methods) (67, 68) were deemed to interfere with downstream DNA amplification (see Text S1 and Fig. S1 in the supplemental material). To create a cell extract with the “gentle” method, 10 mM EDTA (final concentration) was added to the sample tubes containing thawed rock pieces in 1× GlyTE buffer followed by physical disruption by shaking on a vortex at setting 7 (model Vortex-Genie-2) for 5 min and 1 min of sonication of the sample while on ice. Rock particles were pelleted by centrifuging at 1,000 rpm, and the remaining supernatant centrifuged at 10,000 rpm in order to pellet cells suspended in liquid. Pelleted cells were resuspended in 1 ml of GlyTE. A detailed step-by-step protocol is found on Protocols.io with DOI number dx.doi.org/10.17504/protocols.io.bvrmn546).

The cell extracts were thawed, mixed with SYTO 9 Green Fluorescent Nucleic Acid stain (Thermo Fisher Scientific; 5 μM final concentration) and passed through a 40-μm-mesh nylon filter prior to FACS. Cells were sorted in a clean room within the Single Cell Genomics Center (SCGC) located at the Bigelow Laboratory for Ocean Sciences using a Becton Dickinson (formerly Cytopeia) Influx Mariner with sterile 15 ppt NaCl solution as sheath fluid (69). For bulk concentration of cells for screening, collections of 1,000 cell-like particles were sorted into a 1.5-ml centrifuge tube containing a 1-μl landing pad of 1× GlyTE using a blue solid-state laser (488 nm) line for excitation. The sort gate was manually set based on red (692/40) and green (531/40) relative fluorescence (example flow cytogram provided in Fig. S2). Sorted cells were physically lysed (five freeze-thaw cycles) before chemical lysis, neutralization, and multiple displacement amplification (MDA) with the REPLI-g minikit (Qiagen, Valencia, CA) according to the manufacturer’s protocol. Dilutions of amplified DNA (0, 1:10, 1:100) were used to verify a 16S rRNA gene product via PCR with universal 518F and 800R primers (518F, CCAGCAGCCGCGGTAATACG; 800R, TACCAGGGTATCTAATCC) (70) prior to sequencing, with the most successful dilution of DNA treated with the REPLI-g minikit used for downstream amplicon sequencing. Negative REPLI-g amplification controls were included with every batch of samples.

10.1128/mBio.00490-21.4 FIG S2 Taxonomic identify of sorted single cells from sediment sample 357-69A-4R1-5.41 mbsf. The colored symbols represent particles that were sorted based on red (*x* axis) and green (*y* axis) fluorescence emission, using 692/40 nm and 531/40 nm band pass filters, respectively. Particles were excited with a 488 nm laser. The smaller gray symbols represent particles that were detected but not sorted. See Data Set S1 for more detailed taxonomic information. Download FIG S2, DOCX file, 2.1 MB. Copyright © 2021 Goordial et al. 2021 Goordial et al. https://creativecommons.org/licenses/by/4.0/ This content is distributed under the terms of the Creative Commons Attribution 4.0 International license.

### DNA sequencing and analysis.

Illumina-based amplicon sequencing of the 16S rRNA gene V4V5 hypervariable region from the bulk cell sort amplified DNA was done at the Marine Biological Laboratory (MBL) (Woods Hole, MA, USA) as part of the Deep Carbon Observatory’s Census of Deep Life, as described in the supplemental material. Additional amplicon sequencing was done at the Integrated Microbiome Resource at Dalhousie University. REPLI-g amplification-negative controls were pooled and included with every sequencing batch. The sequence data sets were analyzed using DADA2 (71) for classification and generation of amplicon sequence variants (ASVs) and *mothur* v.1.39.5 (72) for grouping sequences into operational taxonomic units (OTUs) of ≥97% sequence similarity, as described in the supplemental material. Additionally, comparative analyses of ASVs with other sequence data from IODP Expedition 357 were carried out to determine the proportion of sequences that may have sourced from contamination (supplemental material). We compared our amplicon sequence data to those of Motamedi et al. (27) which explicitly examined the potential for contamination in Atlantis Massif subsurface samples through amplicon analyses. This included 212 amplicon data sets derived from water column samples collected during the IODP X357 drilling expedition, as well as 14 drilling grease samples. The analyses carried out in Motamedi et al. (27) generated a data set of 684 ASVs considered to be “Likely Indigenous” microbial sequences by carrying out differential abundance and simple overlap analyses on their larger data set. The cores from the Motamedi study were collected during the same research expedition, and subsamples split for analyses by separate lab. ASV sequences that were identified as 99% similar to those in the no-template controls, or in the grease and water samples from the Motamedi study were considered to be contaminants. An exception to this was if such reads were also identified in the Motamedi study as likely to be indigenous via their methods and larger data set. In such instances, we examined the environmental source of the closest BLAST match (see Table S2 in the supplemental material). Based on this information, the ASV was manually assigned as either possibly subsurface or likely contaminant. This resulted in grouping sequences in four categories of sequence origin: subsurface, possibly subsurface, likely contamination, and contamination.

Based on diversity in 16S rRNA gene amplicon sequence screens, one sediment and three crustal samples were chosen for additional metagenomic sequencing at the MBL (highlighted in bold in Table 1). DNA sequencing, analysis, and metagenome assembly methods are described in detail in the supplemental material. Metagenome-assembled genomes (MAGs) were annotated using Prokka (73), the Joint Genome Institute (JGI) Integrated Microbial Genomes & Microbiomes (IMG/M) pipeline (74), and GhostKoala (75). Genes of interest in the MAGs were searched for using the python script dna_analysis.py (https://github.com/JackieGOO/dna-profiling). A heatmap of completeness of pathways (based on proteins unique to that pathway) and proteins of interest was made using KEGG annotated genes from GhostKoala, and the python script KEGG-decoder.py (76) with some modification; the altered python script and all other scripts used can be found at https://github.com/JackieGOO/AMRocks.

The generation and low-coverage genomic sequencing of single-cell amplified genomes (SAGs) from one sediment-basement interface sample (sample 357-69A-4R1-5.41 m below seafloor [mbsf]) was performed at the SCGC with a protocol described previously (77). Briefly, cell-like particles were individually sorted into 384-well plates, followed by cell lysis and whole-genome amplification using the WGA-X DNA polymerase, and whole-genome sequencing, genome assembly, and annotation using Prokka carried out as described in reference 77. Multiple approaches were used to classify the taxonomy of the resulting SAGs and was similar to the steps carried out as for MAGs as described in the supplemental material.

Phylogenomic trees were created using concatenated genes from abundant taxa grouping in the Subsurface and Possibly Subsurface categories to further examine the potential for these taxa to represent contaminants as described in the Supplemental Methods. SAG and MAG contigs were run through VirSorter (78) (version 1.0.5, using the “--db 2” option) to identify potential viruses. Contigs and contig regions falling into VirSorter categories 1 and 2 were considered viral.

### Data availability.

16S rRNA gene amplicon data sets are available in NCBI SRA under project numbers PRJNA610843 and PRJNA590516. Metagenomic data sets are available at the Joint Genome Institute’s IMG/MER database under accession numbers 3300031850 (sample 357-70C-3R1-3.55 mbsf), 3300031906 (sample 357-69A-4R1-5.41 mbsf), 3300031907 (sample 357-69A-9R2-14.61 mbsf), and 3300022577 (sample 357-68B-1R1-1.44 mbsf). Sanger sequenced 16S rRNA genes from single-cell genomes from sediment sample 357-69A-4R1-5.41 mbsf are available on SRA under accession numbers MN715239 to MN715312. SAG and MAG data sets are available at the Joint Genome Institute’s IMG/MER under GOLD Study identifier (ID) Gs0136008 and Gs0133404, respectively. Consensus ID, genome statistics for all MAGs and SAGs, and their accession numbers on IMG are provided in Data Set S1.
