# Supplementary material for: Microbial Diversity and Function in Shallow Subsurface Sediment and Oceanic Lithosphere of the Atlantis Massif
Source: mBio. 2021 Aug 3;12(4):e00490-21. doi: 10.1128/mBio.00490-21 (PMC8406227; doi:10.1128/mBio.00490-21)
Supplement: TEXT S1 [file mbio.00490-21-t0001.docx]

**Supplementary methods:**

**Evaluation of cell extraction methods from environmental samples**

Multiple chemical and physical treatments to aid cell extraction were tested on basalt samples from Integrated Ocean Drilling Program (IODP) Expedition 336 Mid-Atlantic Ridge Microbiology basalt samples from site U1383 (Table S2), and submerged coastal rock samples from Bigelow Laboratory for Ocean Science’s dock (Figure S1), with a focus on determining how these methods impacted the ability to amplify DNA from fluorescence-activated cell sorting (FACS). Multiple “harsh” treatments based on cell extractions used for quantitative cell extraction from sediment were tested (Kallmeyer et al 2008, Morono et al 2013), alone or in combination: 3% NaCl, 2M oxalic acid, 0.02% hydrochloric acid, detergents (0.1% of each Tween 20 and 80) and 10 mM sodium metatungstate. Cells were then separated from the sediment/rock particles using a density gradient with 80% Nycodenz or Percoll (Table S2) according to the protocol of Kallmeyer et al, 2008 with the modification of omitting cell fixation steps to permit downstream PCR amplification tests. Different mixing devices were also tested, a sonicator bath JSP Ultrasonic Cleaner, or vortex. A “gentle” method, with 10 mM EDTA, was also tested. FACS was used to sort 1,000 cell-like particles to test the effects of these treatments on DNA amplification with the same REPLI-g kit used as described in the main text. While cells were liberated in multiple treatments, the “Gentle” protocol that only included physical disruption in a salt/EDTA buffer was the only one that resulted in amplifiable DNA (Table S2).

To assess the proportion of cells liberated using the “gentle” protocol in comparison to a “harsh” protocol, we conducted tests using granite rock samples collected from coastal Gulf of Maine close to Bigelow Laboratory for Ocean Sciences. “Gentle” cell extractions (in triplicate) were carried out as described for oceanic crust samples, in addition to a “harsh” density gradient method that utilized physical and chemical disruption as well as density gradient cell concentration (Kallmeyer et al 2008, Morono et al 2013). In addition, the “harsh” extraction was carried out on rock samples after the “gentle” extraction had been carried out, to see what fraction of cells remained. Cells from all tests were stained for ten minutes in the dark using SYTO green DNA stain (Thermofisher) as per manufacturers instruction (final concentration 1X) and quantified via flow cytometry, as described below. These tests revealed that the “gentle” extraction method recovered an order of magnitude fewer cells than the total cells that could be extracted with the “harsh” method (Figure S1). Thus, interpretations of community structure based on the “gentle” extraction approach should be viewed through this lens.

**Amplicon DNA sequencing and analysis.**

DNA was sent for amplicon sequencing to two centers. Four DNA extracts were sequenced at the Marine Biological Laboratory Bay Paul Center (MBL; 69A-9R2-14.61mbsf, 70C-3R1-3.55mbsf, 69A-4R1-5.91mbsf, and 74A-1R1-0.5mbsf, Table S1), and the remaining four (69A-4R1-5.41 mbsf-mini, 69A-4R1-5.41 mbsf-sc, 68B-7R1-7.73mbsf, and 68B-3R1-3.8mbsf) were sent to the Integrated Microbiome Resource facility at Dalhousie University (Halifax, Canada). Negative controls consisting of pooled Repli-G negative controls were sent for sequencing with every batch. At the MBL, the V4-V5 hypervariable regions of the bacterial 16S rRNA gene were amplified using degenerate primers. The primers targeting Bacteria were 518F (CCAGCAGCYGCGGTAAN) and 926R (a mixture of CCGTCAATTCNTTTRAGT, CCGTCAATTTCTTTGAGT, and CCGTCTATTCCTTTGANT). Amplification was done with fusion primers containing the 16S-only sequences fused to Illumina adapters. The pool was sequenced on an Illumina MiSeq in a paired-end sequencing run using dedicated read indexing. Primers targeting the Archaeal V4-V5 region were (517F – GCCTAAAGCATCCGTAGC, GCCTAAARCGTYCGTAGC, GTCTAAAGGGTCYGTAGC, GCTTAAAGNGTYCGTAGC, GTCTAAARCGYYCGTAGC, 958R- CCGGCGTTGANTCCAATT); however, no Archaea were recovered. At IMR, universal V4-V5primers 515F- GTGYCAGCMGCCGCGGTAA and 926R = CCGYCAATTYMTTTRAGTTT were used (Parada et al 2016) and samples sequenced on an Illumina MiSeq in a paired end sequencing run as described elsewhere (Comeau et al 2017). Sequence reads were analyzed using *mothur* v.1.39.5 (Kozich et al 2013) for classification and grouping sequences into Operational Taxonomic Units (OTUs) of ≥97% sequence similarity. Scripts can be found at <https://github.com/JackieGOO/AMrocks>. Briefly, sequences were quality-filtered by removing primer sequences, paired reads < 400 bp long, sequences with ambiguous base calls, and homopolymer repeats greater than 8 bp. Bacterial and archaeal sequences were reduced to include only unique sequences and aligned to the *mothur*-interpreted Silva database containing unique sequences from the SSU Ref database (v.132) and trimmed to equal size. Chimera removal using chimera.uchime within *mothur* was used to further reduce sequencing error prior to clustering. OTUs were clustered using average-neighbour clustering with a 97% cut-off. All sequences were classified in *mothur* with the Silva SSU database v. 132 and a 0.80 cut-off (Glöckner et al 2017).

**Identification of amplicon data as potential contaminants**

To identify potential contaminants in the amplicon data, ASVs were generated from the sequences derived in this study using the DADA2 standard pipeline (Callahan et al., 2016) (SOP referenced 6/17/20). Samples sequenced at MBL were truncated at 250 bp for forward reads and 200 bp for reverse reads, and had the primers removed. Samples sequenced at IMR were truncated at 300 BP for forward reads and 250 bp for reverse reads and had primers removed. Both datasets were assigned taxonomy using the naive bayesian classifier in the DADA2 package trained on training set from the Silva v138 ribosomal database. These two datasets were clustered together at the 99% ID using usearch (cluster_fast option) in order to merge the ASVs derived from different primer sets. ASV sequence data from 212 water column and serpentinite rock samples, and 14 drilling grease samples from Motamedi et al., 2020 were downloaded and used for comparative analyses. In addition, 684 ASVs identified to be “Likely Indigenous” by Motamedi et al., 2020 using differential abundance methodology were used for comparison. As these ASVs used different primer sets, these data were clustered to the 99% level as above to the ASVs produced in this study. The cores from the Motamedi study were collected during the same research expedition, and subsamples split for analyses by separate lab groups in a clean room at the Kochi core Center in Japan. DNA extractions and sequencing from the Motamedi study were carried out entirely in a different laboratory in Salt Lake City, Utah, and thus similarities in ASVs based on DNA extraction or sequencing contamination can be ruled out. ASV generated in this study that were identified as 99% similar to those in the no template controls, or in the grease and water samples from the Motamedi study were considered to be contaminants. An exception to this was if such reads were also identified in the Motamedi study as likely to be indigenous via their methods and larger dataset. In such instances, we examined the environmental source of the closest BLAST match (**Table S2**). Based on this information, the ASV was manually assigned as either possibly subsurface, or probable contaminant.

**Metagenomic sequencing and analysis**

From the same amplified DNA samples used above, after shearing the samples to 400 bp using a Covaris, the Nugen Ovation Ultralow Library System v2 kit was used to add adapters and amplify the product for metagenomic sequencing at MBL. The Illumina NextSeq was used to sequence the final prepared libraries. Quality control was carried out using Trimmomatic v 0.36 (Bolger et al 2014). Default settings were used: removed leading and tailing low quality and N bases, trimmed reads when quality per base dropped below 15 with a 4-base window, and discarding reads with length <36 bases. Reads with no mate pair were removed. Trimmed reads were kmer depth normalized using BBNORM from the BBTools suite v 35.85 (https://sourceforge.net/projects/bbmap/) using target=30, min=3, kmer=21. Resulting reads were assembled using SPAdes v 3.9.0 (Bankevich et al 2012), using the --meta flag for metagenomic assembly, with kmer selection of 21, 33, and 55. Reads were mapped using BBMAP from BBTools, and binned using Metabat2 (Kang et al 2019) (with a minimum contig length of 1500 bp). Metagenome assembled genomes (MAGs) were annotated using IMG/MER Prokka (and GhostKoala for KEGG maps). Genes of interest in the MAGs were searched for using the python script dna_analysis.py (https://github.com/JackieGOO/AMrocks), and select proteins of interest manually searched against the GenBank non-redundant database. A heatmap of completeness of pathways and proteins of interest was made using KEGG annotated genes from GhostKoala, and the python script KEGG-decoder.py (Graham et al 2018).

**Low coverage SAG classification**

Multiple approaches were used to classify the taxonomy of individual SAGs **(supplementary file 1):** Sanger sequencing of the 16S rRNA gene (primers 27F: AGR GTT YGA TYM TGG CTC AG, and 907R: CCG TCA ATT CMT TTR AGT TT), and various pipelines applied to assembled SAG data including CheckM, and checkM’s SSU_finder command (https://github.com/Ecogenomics/CheckM/), the Genome Taxonomy database (GTDBK) release 86 (https://github.com/Ecogenomics/GTDBTk), and the Microbial Genome Atlas, MiGA (accessed April, 2019, http://microbial-genomes.org/). If SSU data were available for a SAG via Sanger sequencing or SSU_finder, these data were used to search the Silva database (SINA 1.2.11; https://www.arb-silva.de/aligner/) and this taxonomy was used for classification to the highest resolution available. If an SSU sequence was not found, the GTDBK taxonomy was used on assembled SAG data, but the output converted to the same taxonomy structure used by Silva to the class level; if a direct comparison could not be determined, the resolution was kept to the phylum level to remain congruent. For genomes in which CheckM and MiGA were the only methods to assign taxonomy, the level of resolution was kept where both methods were congruent. If found incongruent, the CheckM assigned taxonomy was used, but only at the phylum level. If CheckM was unresolved, but MiGA provided a classification, the MiGa classification was used to the phylum level.

**Phylogenetic Tree and unclassified sequences**

Unclassified OTUs from the top 100 most numerically abundant OTUs were taken from the whole dataset (n=39). These OTUs were aligned to the Silva NR database via the online ACT tool (Pruesse et al 2012). A preliminary phylogenetic tree showed that 30/39 of these OTUs formed a clade that grouped with environmental sequences annotated to the phylum Acidobacteria. Those 30 OTUs were taken as a subset for a more thorough phylogenetic analysis. These sequences were aligned to the NR database, and neighbors (maximum five per sequence) with a minimum identity of 80% ID were exported. This resulted in an alignment with 77 sequences. The resulting fasta file was converted to phylip format and trimmed to the regions of the amplified OTU sequences (367 base pairs). Tree topology inference and bootstrapping was performed using RAXML with the GTRCAT evolution model and 1,000 bootstrap replicates. Trees were imported into iTOL (Letunic and Bork 2016) for visualization and annotation, and branches with no OTUs or SAGs sequences removed.

**Phylogenetic Analyses of MAGs and SAGs**

Phylogenetic trees of the concatenated conserved proteins of the SAGs and MAGs with members of their respective phyla or classes were constructed to identify neighboring genomes and phylogenetic relationships.  For the trees, all genomes from the given phyla or class were downloaded from IMG and NCBI and duplicate entries were removed. This analysis was performed using the standard workflow of PhyloPhlan 3.0 (Asnicar et al., 2020).  The AMPHORA2 conserved single-copy protein set described in Wu and Scott 2012 was used.  Trees were constructed using RaXML (version 8, Stamatkis, 2014) using the PROTCATLG model with 100 bootstraps and a most likelihood search after bootstrapping (Le and Gascuel et al., 2008). The trees were then manually annotated to include the environment that the genome or isolate was derived from by manually searching individual accession numbers on GenBank or IMG.

**Supplementary References**

Asnicar, F., Thomas, A. M., Beghini, F., Mengoni, C., Manara, S., Manghi, P., Zhu, Q., Bolzan, M., Cumbo, F., May, U., Sanders, J. G., Zolfo, M., Kopylova, E., Pasolli, E., Knight, R., Mirarab, S., Huttenhower, C., & Segata, N. (2020). Precise phylogenetic analysis of microbial isolates and genomes from metagenomes using PhyloPhlAn 3.0. Nature Communications, 11(1), 1–10.

Bankevich A, Nurk S, Antipov D, Gurevich AA, Dvorkin M, Kulikov AS *et al* (2012). SPAdes: a new genome assembly algorithm and its applications to single-cell sequencing. *Journal of computational biology* **19:** 455-477.

Bolger AM, Lohse M, Usadel B (2014). Trimmomatic: a flexible trimmer for Illumina sequence data. *Bioinformatics* **30:** 2114-2120.

Callahan, B. J., P. J. McMurdie, M. J. Rosen, A. W. Han, A. J. A. Johnson and S. P. Holmes (2016). "DADA2: high-resolution sample inference from Illumina amplicon data." Nature methods 13(7): 581-583.

Comeau AM, Douglas GM, Langille MGI (2017). Microbiome Helper: a Custom and Streamlined Workflow for Microbiome Research. *mSystems* **2:** e00127-00116.

Früh-Green GL, Orcutt BN, Rouméjon S, Lilley MD, Morono Y, Cotterill C *et al* (2018). Magmatism, serpentinization and life: Insights through drilling the Atlantis Massif (IODP Expedition 357). *Lithos*.

Glöckner FO, Yilmaz P, Quast C, Gerken J, Beccati A, Ciuprina A *et al* (2017). 25 years of serving the community with ribosomal RNA gene reference databases and tools. *Journal of Biotechnology* **261:** 169-176.

Graham ED, Heidelberg JF, Tully BJ (2018). Potential for primary productivity in a globally-distributed bacterial phototroph. *The ISME Journal* **12:** 1861-1866.

Kallmeyer J, Smith DC, Spivack AJ, D'Hondt S (2008). New cell extraction procedure applied to deep subsurface sediments. *Limnology and Oceanography: Methods* **6:** 236-245.

Kang D, Li F, Kirton ES, Thomas A, Egan RS, An H *et al* (2019). MetaBAT 2: an adaptive binning algorithm for robust and efficient genome reconstruction from metagenome assemblies. *PeerJ Preprints* **7:** e27522v27521.

Kozich JJ, Westcott SL, Baxter NT, Highlander SK, Schloss PD (2013). Development of a dual-index sequencing strategy and curation pipeline for analyzing amplicon sequence data on the MiSeq Illumina sequencing platform. *Appl Environ Microb* **79:** 5112-5120.

Letunic I, Bork P (2016). Interactive tree of life (iTOL) v3: an online tool for the display and annotation of phylogenetic and other trees. *Nucleic acids research* **44:** W242-W245.

Morono Y, Terada T, Kallmeyer J, Inagaki F (2013). An improved cell separation technique for marine subsurface sediments: applications for high‐throughput analysis using flow cytometry and cell sorting. *Environmental microbiology* **15:** 2841-2849.

Orcutt BN, Bergenthal M, Freudenthal T, Smith D, Lilley MD, Schnieders L *et al* (2017). Contamination tracer testing with seabed drills: IODP Expedition 357. *Scientific Drilling* **23:** 39-46.

Parada AE, Needham DM, Fuhrman JA (2016). Every base matters: assessing small subunit rRNA primers for marine microbiomes with mock communities, time series and global field samples. *Environmental Microbiology* **18:** 1403-1414.

Pruesse E, Peplies J, Glöckner FO (2012). SINA: Accurate high-throughput multiple sequence alignment of ribosomal RNA genes. *Bioinformatics* **28:** 1823-1829.

Si Quang Le, Olivier Gascuel, An Improved General Amino Acid Replacement Matrix, Molecular Biology and Evolution, Volume 25, Issue 7, July 2008, Pages 1307–1320,

Stamatakis, A. (2014). RAxML version 8: a tool for phylogenetic analysis and post-analysis of large phylogenies. Bioinformatics **30**(9): 1312-1313.

Wu, M., & Scott, A. J. (2012). Phylogenomic analysis of bacterial and archaeal sequences with AMPHORA2. Bioinformatics, 28(7), 1033–1034.
