# Supplementary material for: Microbial Diversity and Function in Shallow Subsurface Sediment and Oceanic Lithosphere of the Atlantis Massif
Source: mBio. 2021 Aug 3;12(4):e00490-21. doi: 10.1128/mBio.00490-21 (PMC8406227; doi:10.1128/mBio.00490-21)
Supplement: FIG S4 [file mbio.00490-21-sf004.docx]

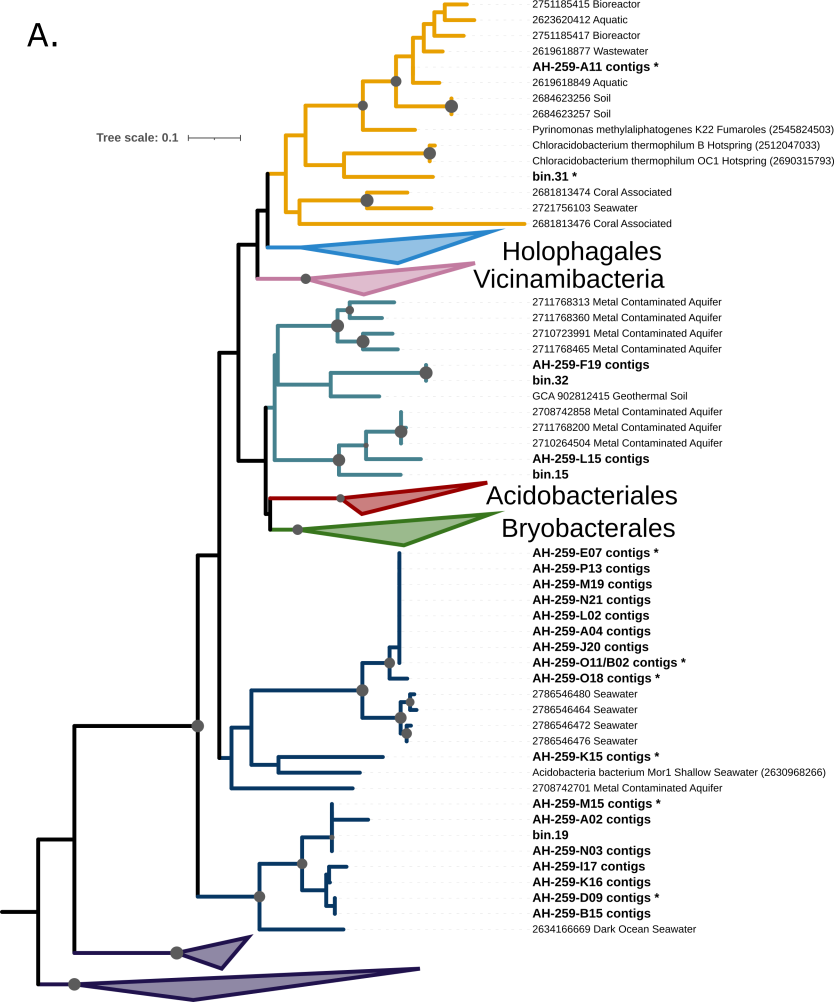


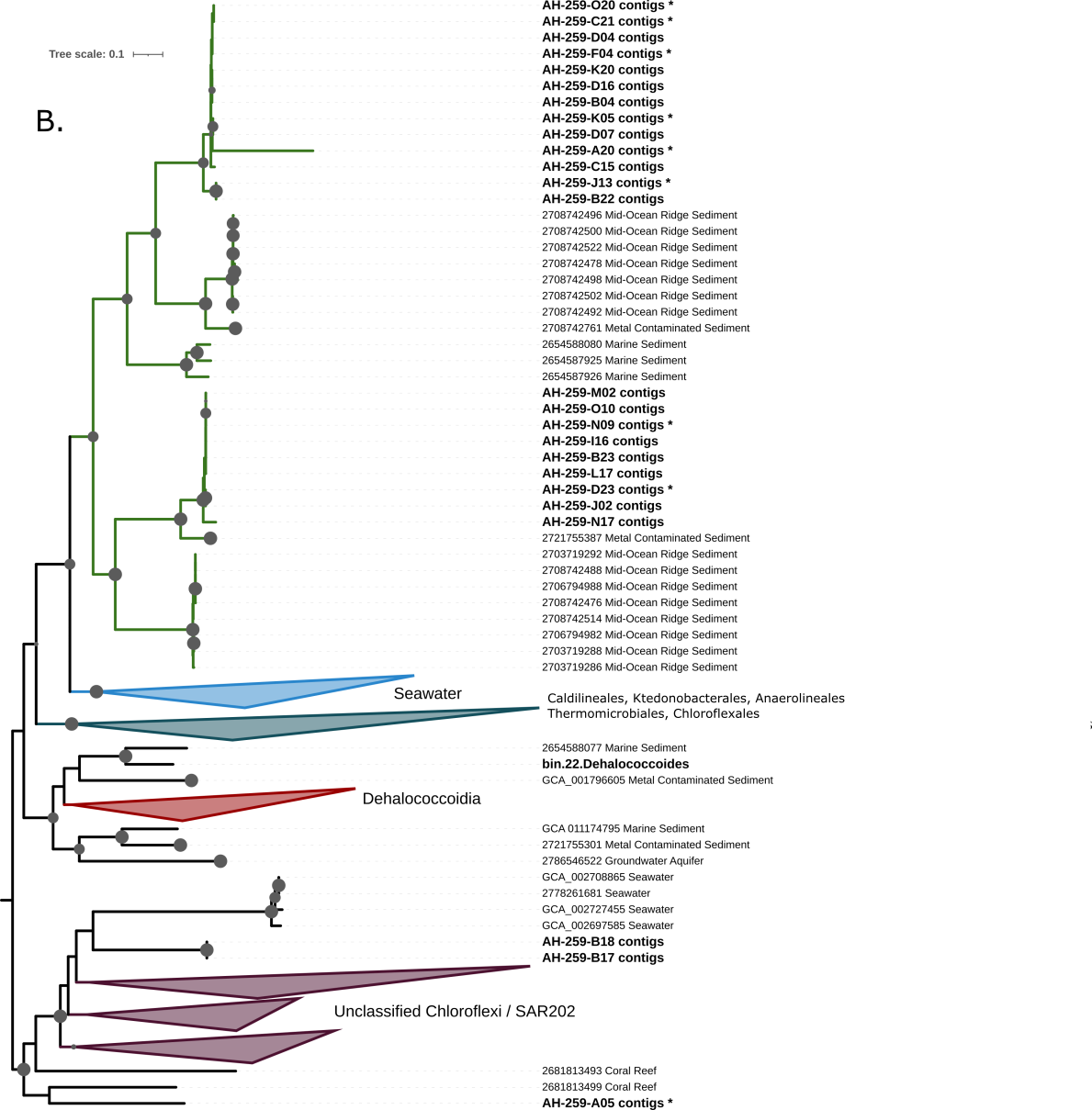


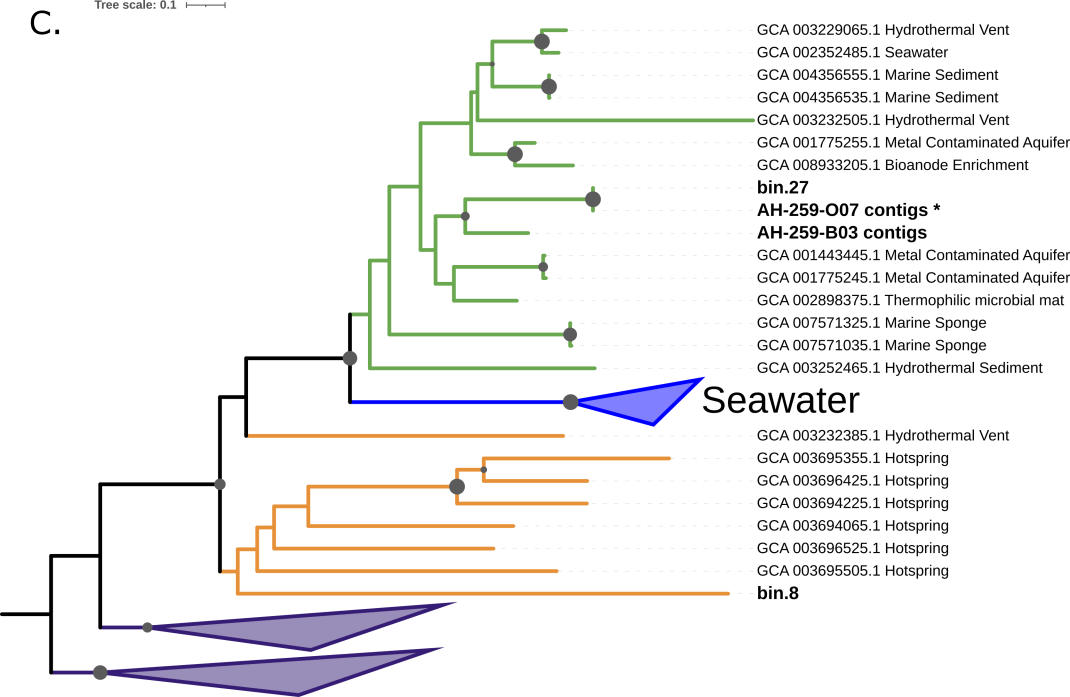


**Figure S4. Phylogenomic placement of MAGs and SAGS supports subsurface origin of Acidobacteria (A), Chloroflexi (B), Dadabacteria (C)) taxa.** MAGs from this study labelled as “bins”, SAGs generated in this study labelled with SAG ID with prefix AH-259. Other related genomes are labelled with GenBank or IMG accession number and a description of the environment that the isolate or metagenomic DNA was obtained from. Collapsed clades are at the order level of pure culture isolates within those clades. Collapsed clades that have an environment as a title contain no isolate genomes. Acidobacteria and Dadabacteria are rooted by Aquificales (purple clades), and Chloroflexi is rooted at the mid-point with no outgroup. MAGs with less than five identified marker genes for concatenation are denoted with an *.
