# Supplementary material for: Microbial Diversity and Function in Shallow Subsurface Sediment and Oceanic Lithosphere of the Atlantis Massif
Source: mBio. 2021 Aug 3;12(4):e00490-21. doi: 10.1128/mBio.00490-21 (PMC8406227; doi:10.1128/mBio.00490-21)
Supplement: TABLE S1 [file mbio.00490-21-st001.docx]

**Supplemental Table S1. Numbers of OTUs in amplicon surveys.** Summary of Illumina amplicon sequencing of bulk cell sorted samples from IODP Expedition 357, indicating total number of paired-end (PE) sequence reads and number of estimated operational taxonomic units (OTUs, calculated at 97% sequence similarity or higher, without subsampling to smallest sample size). One sample was bulk cell sorted into two tubes and MDA reaction was performed with two different REPLI-g kits (mini kit and single cell kit). One negative control from the MDA reactions was also included to assess for possible sequence contamination.

| **Hole-Core Section-Depth (mbsf)** | **No. of PE sequence reads** | **No. of OTUs** |
| --- | --- | --- |
| 68B-3R1-3.8 | 71,944 | 150 |
| 68B-7R1-7.73 | 35,954 | 140 |
| 69A-4R1-5.41 (mini kit) | 59,650 | 199 |
| 69A-4R1-5.41 (Single Cell kit) | 49,954 | 386 |
| 69A-4R1-5.91 | 584,461 | 1,928 |
| 69A-9R2-14.61 | 123,442 | 1,838 |
| 70C-3R1-3.55 | 88,153 | 857 |
| 74A-1R1-0.5 | 236,781 | 1,434 |
| Negative control 1 | 146 | 36 |
| Negative control 2 | 306 | 85 |
