# Supplementary material for: Microbial Diversity and Function in Shallow Subsurface Sediment and Oceanic Lithosphere of the Atlantis Massif
Source: mBio. 2021 Aug 3;12(4):e00490-21. doi: 10.1128/mBio.00490-21 (PMC8406227; doi:10.1128/mBio.00490-21)
Supplement: FIG S1 [file mbio.00490-21-sf001.docx]

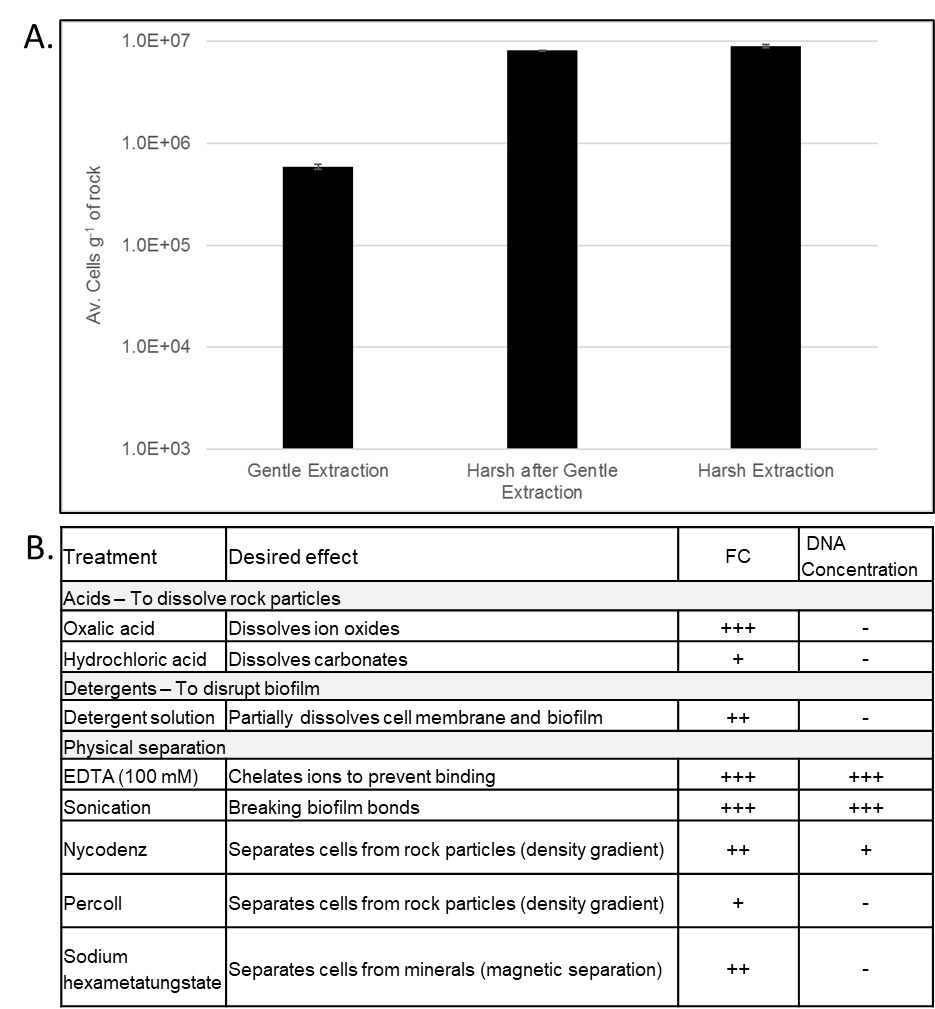


**Supplemental Figure S1. Evaluation of cell separation methodology. (**A) Cell quantification in test rock samples via flow cytometry in samples after “Gentle” cell extraction using salt buffers, a “Harsh” cell extraction using a Nycodenz density gradient solution (as described by (Morono et al 2013)), and a harsh cell extraction on samples after the gentle extraction had been carried out. (B) Effect of different cell separation methods on flow cytometry (FC) sorting and DNA amplification. For flow cytometry assessment column, more “+” symbols indicates qualitatively more cell-like particles observed during FACS. For DNA concentration assessment column, symbols indicate relative amount of DNA quantified in sample after whole genome amplifiation: ‘+++’ :>600 ng/µl, ‘+’ 10-100 ng/µl, ‘-‘: <5 ng/µl.
