# Supplementary material for: Microbial Diversity and Function in Shallow Subsurface Sediment and Oceanic Lithosphere of the Atlantis Massif
Source: mBio. 2021 Aug 3;12(4):e00490-21. doi: 10.1128/mBio.00490-21 (PMC8406227; doi:10.1128/mBio.00490-21)
Supplement: FIG S3 [file mbio.00490-21-sf003.docx]

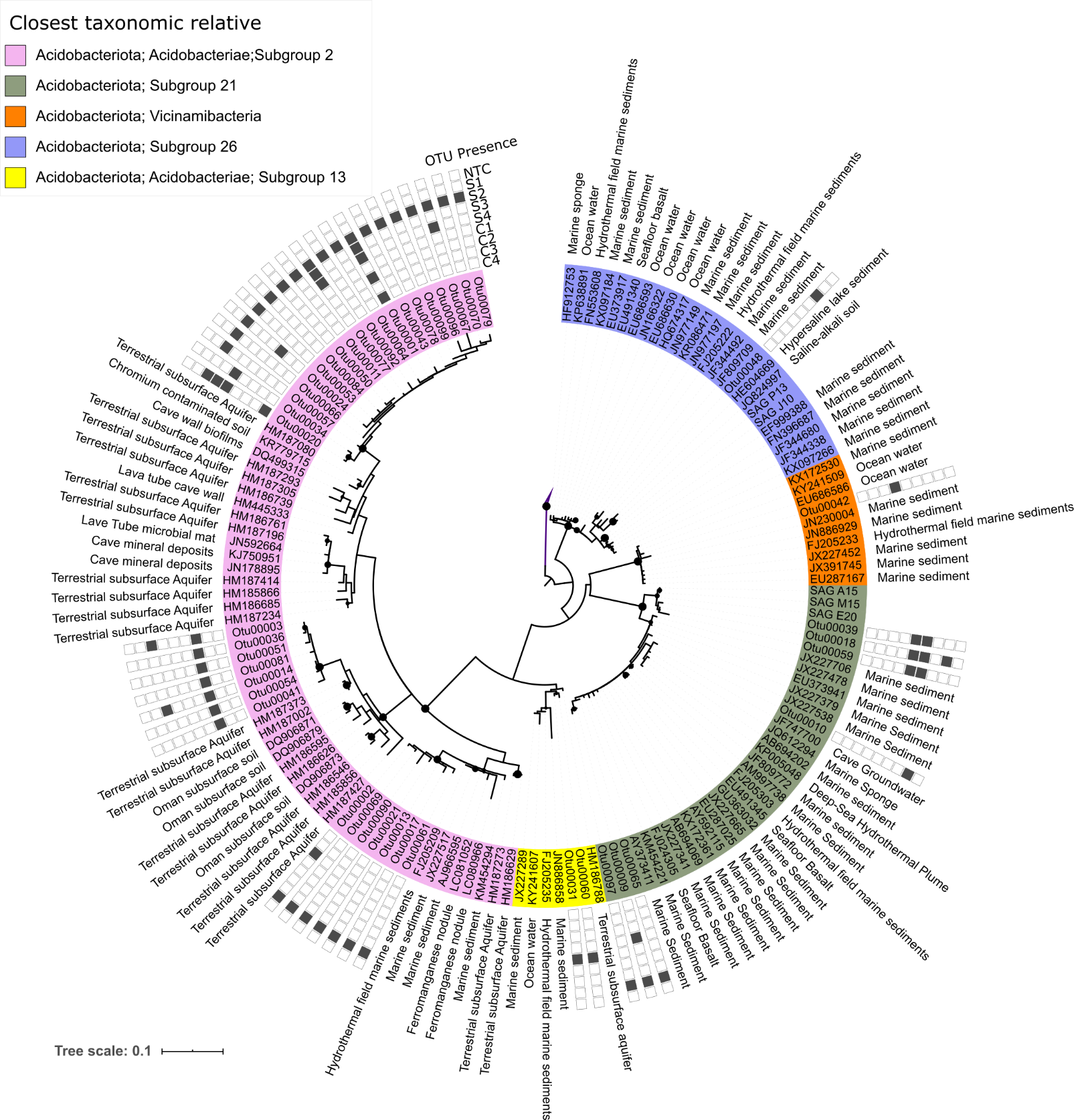


**Figure S3. Phylogenetic placement of unclassified bacterial OTUs with the Acidobacteria phyla from 16S rRNA gene amplicon sequencing of Atlantis Massif sorted cells.** Bootstrap values supporting branching order of phylogenetic tree are displayed as circular symbols at nodes, with no symbols on nodes that have a bootstrap value of less than 50. Collapsed branch consists of Proteobacterial sequences as an outgroup. Inner ring provides the name of the OTU or SAG from this study (see Supplemental File 1) or the NCBI Genbank accession number for closest sequence relatives from published studies, with color denoting Acidobacteria subgroup classification as indicated in the legend. Outer ring indicates the environment type for the closest environmental sequences or the presence (filled)/absence (unfilled) of the OTU in each sample from this study: NTC, No Template control; S1:74A-1R1-0.5mbsf; S2, 69A-4R1-5.91mbsf; S3, 69A-4R1-5.41mbsf (mini MDA kit); S4, 69A-4R1-5.41mbsf (single cell MDA kit); C1, 70C-3R1-3.55mbsf; C2, 69A-9R2-14.61mbsf; C3, 68B-7R1-7.73mbsf; and C4, 68B-3R1-3.8mbsf.
