# Supplementary material for: Microbial Diversity and Function in Shallow Subsurface Sediment and Oceanic Lithosphere of the Atlantis Massif
Source: mBio. 2021 Aug 3;12(4):e00490-21. doi: 10.1128/mBio.00490-21 (PMC8406227; doi:10.1128/mBio.00490-21)
Supplement: TABLE S2 [file mbio.00490-21-st002.docx]

**Supplemental Table S2. Environment of closest blast match of abundant (>5000 reads) amplicon contaminants**

| **ASV** | **ID (Family)** | **Env. Of closest BLAST match (% similarity) [Accession]** | **Assignment in present study** | **Comments** |
| --- | --- | --- | --- | --- |
| AmiAsv_1 | Rhizobiaceae | Soil (100%) [CP044218.1] | Likely Contaminant | Taxonomy suggests contaminant |
| AmiAsv_10 | Rhizobiaceae | Soil (99.74%) [MK621282.1] | Likely Contaminant | Taxonomy suggests contaminant |
| AmiAsv_2 | Unclassified Dadabacteriales (order) | ferromanganese crust  (100%) [LC138801.1] | Possibly Subsurface | Present in low abundance in NTC (2 reads). Closest match suggests subsurface organism. |
| AmcAsv_3 | Unclassified Acidobacteriae Subgroup 2 (order) | deep-sea polymetallic  nodules (99.5%) [JX227517.1] | Possibly Subsurface | Present in Motamedi 2020 water and rock samples, and identified as likely indigenous in rock samples. Closest match suggests subsurface organism. |
| AmcAsv_11 | Halomonadaceae | Salt rocks, China (99.7%) [MT433876.1] | Likely Contaminant | Present in Motamedi 2020 as likely indigenous in rock samples. Present in NTC in high abundance. Taxonomy suggests contaminant. |
| AmcAsv_12 | Idiomarinaceae | Mud Volcano (100%) [EU432584.1] | Likely Contaminant | Taxonomy suggests contaminant. |
| AmcAsv_13 | Idiomarinaceae | biofilm from alkaline ikaite tufa column, Greenland (99.74%) [EU180987.1] | Likely Contaminant | Taxonomy suggests contaminant. Closest matches are from alkaline environments like the Atlantis Massif |
| AmcAsv_19 | Unclassified Acidobacteriota Subgroup 21 (class ) | Bottom water at the Suiyo Seamount (98.2%) [AB629266.1] | Possibly Subsurface | Present in Motamedi 2020 water and rock samples, and identified as likely indigenous in rock samples. Closest match suggests subsurface organism. |
