# Supplementary material for: Microbial Diversity and Function in Shallow Subsurface Sediment and Oceanic Lithosphere of the Atlantis Massif
Source: mBio. 2021 Aug 3;12(4):e00490-21. doi: 10.1128/mBio.00490-21 (PMC8406227; doi:10.1128/mBio.00490-21)
Supplement: FIG S5 [file mbio.00490-21-sf005.docx]

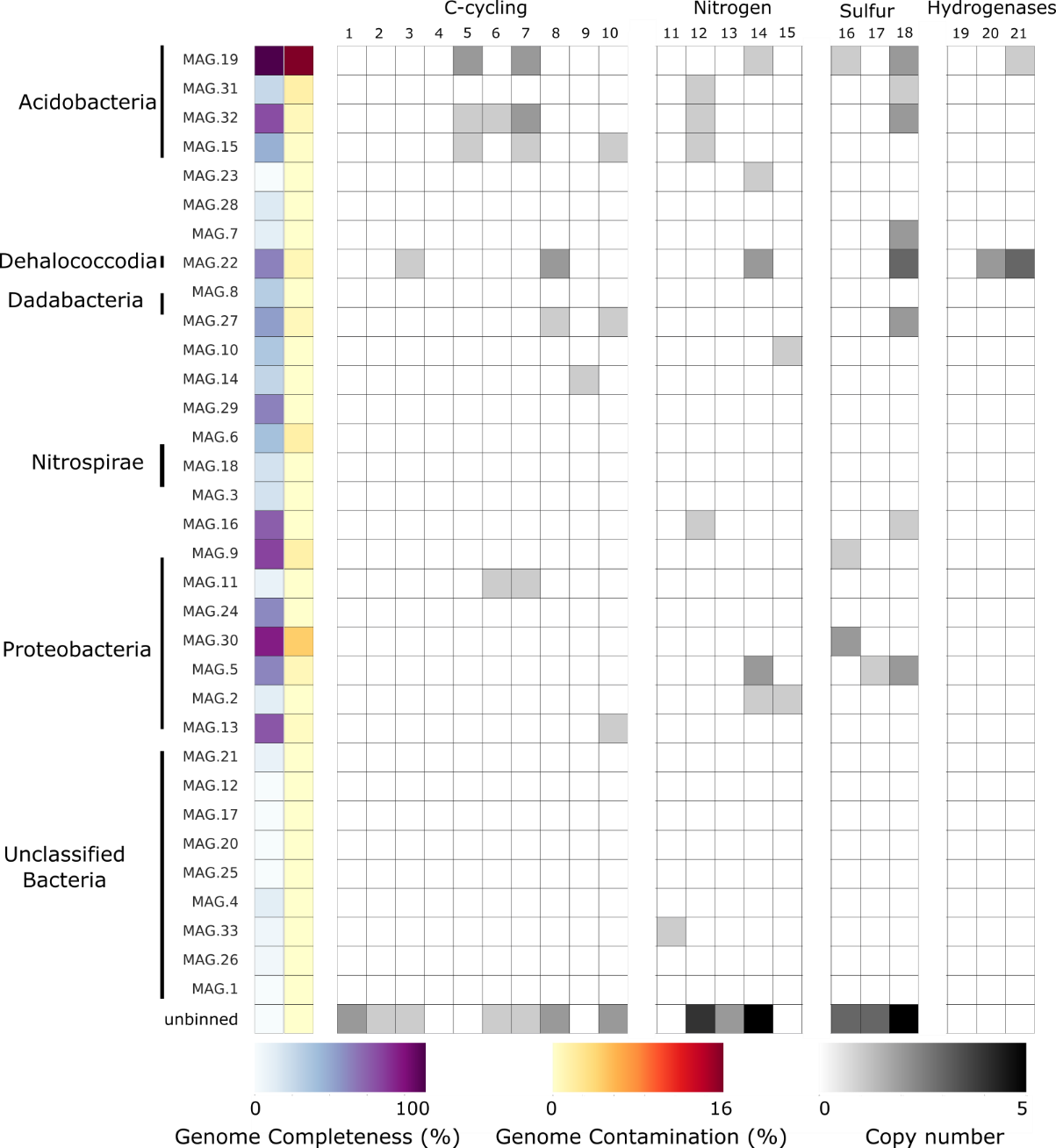


**Figure S5. Expanded view of the metabolic potential of individual Metagenome Assembled Genomes (MAGs).** MAGs grouped on the y-axis by taxonomy. Columns along x-axis: estimated completeness of MAG based on single copy marker genes (scaled from 0-100% per legend), estimated percentage of contamination of genome from checkM (scaled from 0-16% per legend), and copy number of metabolic function genes (scaled from 0-5 copies per legend) grouped as C-cycling pathways [1) RuBisCO, 2) Phosphoribulokiase, 3) Carbon Monoxide Dehydrogenase (CoSH)/Aceteyl-CoA synthase (ACS), 4) ACS, 5) 4-hydroxybutyryl-CoA dehydratase, 6) Methane monooxygenase, 7) isocitrate lyase, 8) CODH large chain, 9) CODH medium chain, 10) CODH small chain], nitrogen cycling pathways [11) ammonia monooxygenase 12) Periplasmic nitrate reductase, 13) Respiratory nitrate reductase, 14) nitrite reductase, 15) nitric oxide reductase], sulfur-cycling pathways [16) adenylylsulfate reductase, 17) phosphoadenosine phosphosulfate reductase, 18)sulfite reductase], and hydrogenases [19) Periplasmic [NiFe] hydrogenase, 20) Periplasmic [NiFeSe] hydrogenase, and 21)NADP-reducing hydrogenase [FeFe]].
