# Supplementary material for: Microbial Diversity and Function in Shallow Subsurface Sediment and Oceanic Lithosphere of the Atlantis Massif
Source: mBio. 2021 Aug 3;12(4):e00490-21. doi: 10.1128/mBio.00490-21 (PMC8406227; doi:10.1128/mBio.00490-21)
Supplement: FIG S2 [file mbio.00490-21-sf002.docx]

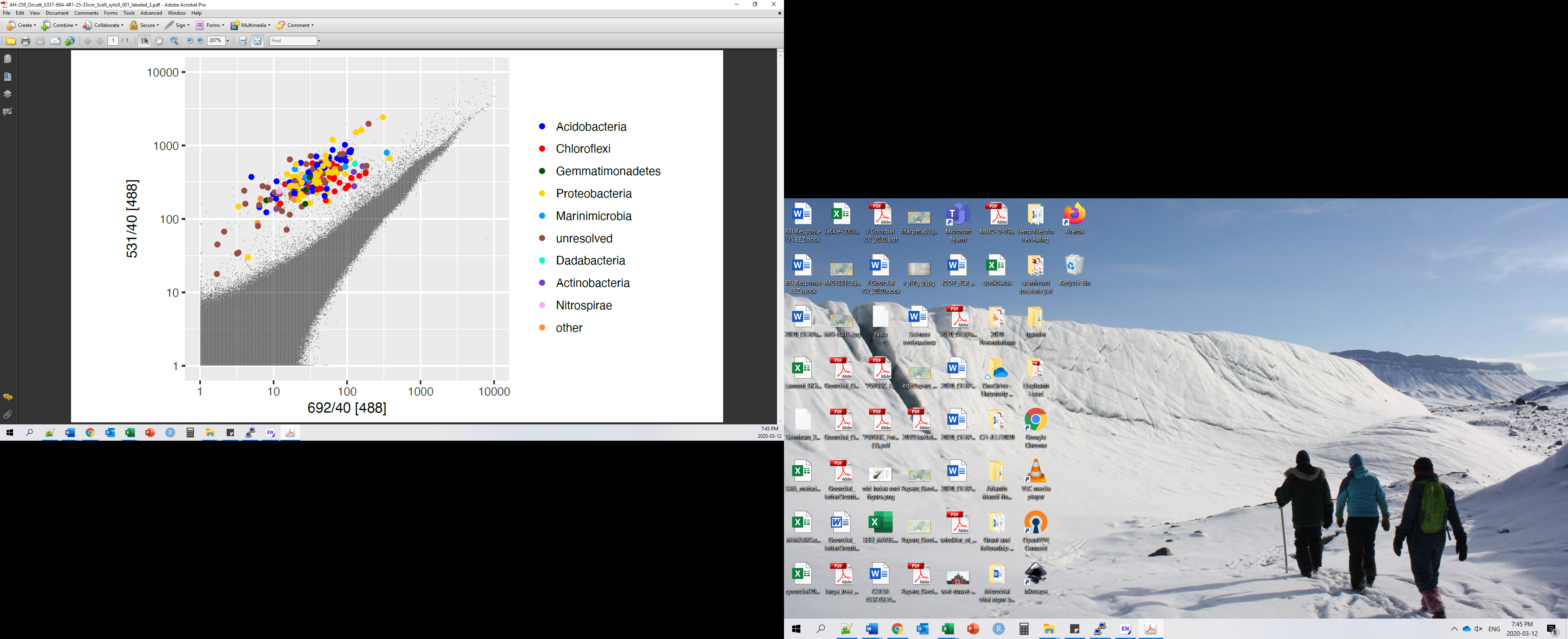


**Supplemental Figure S2. Taxonomic identify of sorted single cells from sediment sample 357-69A-4R1-5.41 mbsf.** The colored symbols represent particles that were sorted based on red (x-axis) and green (y-axis) fluorescence emission, using 692/40 nm and 531/40 nm band pass filters, respectively. Particles were excited with a 488 nm laser. The smaller grey symbols represent particles that were detected but not sorted. See Supplemental File 2 for more detailed taxonomic information.
